# Unraveling the drought-responsive transcriptomes in nodules of two common bean genotypes during biological nitrogen fixation

**DOI:** 10.3389/fpls.2024.1345379

**Published:** 2024-01-26

**Authors:** Helder Anderson Pinto da Silva, Vanessa Santana Caetano, Daniella Duarte Villarinho Pessôa, Rafael Sanches Pacheco, Carlos Henrique S. G. Meneses, Jean Luiz Simões-Araújo

**Affiliations:** ^1^ Programa de Pós-Graduação em Biotecnologia Vegetal, Centro de Ciências da Saúde (CCS), Universidade Federal do Rio de Janeiro (UFRJ), Rio de Janeiro, Brazil; ^2^ Programa de Pós-graduação em Fitossanidade e Biotecnologia Aplicada, Universidade Federal Rural do Rio de Janeiro (UFRRJ), Seropédica, RJ, Brazil; ^3^ Departamento de Biologia, Centro de Ciências Biológicas e da Saúde, Universidade Estadual da Paraíba, Campina Grande, Brazil; ^4^ Centro Nacional de Pesquisa de Agrobiologia, CNPAB – Embrapa, Laboratório de Genética e Bioquímica, Seropédica, RJ, Brazil

**Keywords:** symbiotic nitrogen assimilation, water deficit, genetic transcript profile, RT-qPCR, candidate genes

## Abstract

Common bean (*Phaseolus vulgaris*) can efficiently fix atmospheric nitrogen when associated with Rhizobia. However, drought stress impairs plant metabolic processes, especially the biological nitrogen fixation (BNF). Here, we assessed transcriptional responses in nodules of two common bean genotypes to drought stress under BNF reliance. The RNA-Seq analysis yielded a total of 81,489,262 and 72,497,478 high quality reads for Negro Argel and BAT 477 genotypes, respectively. The reads were mapped to the *Phaseolus vulgaris* reference genome and expression analysis identified 145 and 1451 differentially expressed genes (DEGs) for Negro Argel and BAT 477 genotypes, respectively. Although BAT 477 had more DEGs, both genotypes shared certain drought-responsive genes, including an up-regulated heat shock protein (HSP) and a down-regulated peroxidase, indicating shared pathways activated during drought in nodule tissue. Functional analysis using MapMan software highlighted the up-regulation of genes involved in abiotic stress responses, such as HSPs and specific transcription factors (TFs), in both genotypes. There was a significant down-regulation in metabolic pathways related to antioxidant protection, hormone signaling, metabolism, and transcriptional regulation. To validate these findings, we conducted RT-qPCR experiments for ten DEGs in nodules from both genotypes, for which the expression profile was confirmed, thus reinforcing their functional relevance in the nodule responses to drought stress during BNF. BAT 477 genotype exhibited more pronounced response to drought, characterized by a high number of DEGs. The strong down-regulation of DEGs leads to transcriptional disturbances in several pathways related to stress acclimation such as hormone and antioxidant metabolism. Additionally, we identified several genes that are known to play key roles in enhancing drought tolerance, such as HSPs and crucial TFs. Our results provide new insights into the transcriptional responses in root-nodules, an underexplored tissue of plants mainly under drought conditions. This research paves the way for potential improvements in plant-bacteria interactions, contributing to common bean adaptations in the face of challenging environmental conditions.

## 1 Introduction

Common bean (*Phaseolus vulgaris* L.) is among of the major food crops for direct human consumption, representing the main dietary protein source in developing countries worldwide (Chavez-Mendoza and Sanchez, 2017; Losa et al., 2022). This leguminous species has the ability to symbiotically fix atmospheric nitrogen (N_2_) in association with a specific group of soil diazotrophic bacteria known as rhizobia. In this symbiosis, the rhizobia invade the root tissues and, after a complex molecular dialogue with the host plant, form specialized structures called root-nodules (Cooper, 2007; Ferguson et al., 2010). These symbiotic structures are essential for providing rhizobia with a carbon source and establishing an optimal cellular environment. Within the nodule, rhizobia use the host plant’s energy to convert N_2_ into ammonium (NH_4_^+^) through the action of the bacterial enzyme nitrogenase. This NH_4_^+^ is subsequently absorbed and utilized by the host plant, constituting a fundamental process known as biological nitrogen fixation (BNF) (Masson-Boivin and Sachs, 2018). The BNF has been considered an environmentally friendly technology for plant nitrogen nutrition, sustaining plant growth and development in nitrogen-poor soils. In this case the plants are inoculated with *Rhizobium* strains reducing the use of mineral fertilizers which contributes to satisfactory yield at a low cost (Yoseph and Shanko, 2017). However, seed inoculation technology in the common bean crop has not yet reached its full potential (Hungria and Franco, 1993). These constraints are mainly a result of low nodulation effectiveness in this promiscuous-nodulating host plant, as well as the high susceptibility of symbiosis to abiotic stresses (Hungria and Vargas, 2000; Ramos et al., 2003).

Drought is known as a significant environmental factor that is expected to have an increasingly substantial impact on agricultural regions due to climate change (Feller and Vaseva, 2014; Fahad et al., 2017). It is estimated to affect more than 60% of common bean production worldwide (Beebe et al., 2008; Heinemann et al., 2017). Furthermore, despite the identification of different common bean genotypes that exhibit superior performance under drought conditions (Darkwa et al., 2016; Ribeiro et al., 2019), drought stress significantly impairs BNF in this leguminous species. When common bean plants inoculated with rhizobia are subjected to drought conditions nodule metabolism are disrupted, leading to adverse effects on nitrogenase activity, nitrogen accumulation, and crop yield potential (Mnasri et al., 2007; Devi et al., 2013; Polania et al., 2016), and the impacts on nodule structure and N_2_ fixation, vary among genotypes (Ramos et al., 2003; Devi et al., 2013; Polania et al., 2016). Recent advances on understanding of how drought affects common bean growth have been predominantly based on above-ground traits evaluation (Mukeshimana et al., 2014; Hoyos-Villegas et al., 2017; Wu et al., 2017). In addition, although root-nodules have been used as important sensors of drought-stress in leguminous species grown under symbiosis, the responses of this organ and their drought adaptive mechanisms remain poorly characterized (Kunert et al., 2016). It is known that in peanut nodules under drought-stress conditions nitrogenase activity decreases (Furlan et al., 2014) and amide metabolism is impaired (Furlan et al., 2017). In soybean, drought-stressed nodules exhibit lower water potential (Gil-Quintana et al., 2015) and undergoes to premature senescence (Cilliers et al., 2018) compromising the BNF activity and nitrogen accumulation. Most recently, it has been demonstrated that changes in nitrogenase activity as well as in global gene expression have occurred not only in nodules from *Medicago truncatula* and *Lotus japonicum* in response to drought, but also in their symbiotic partners *Sinorhizobium meliloti* and *Mesorhizobium loti*, which adjust their gene expression in response to water shortage (Sańko-Sawczenko et al., 2019).

The RNA-Sequencing (RNA-Seq) technology has revolutionized the field of plant functional genomics and has accelerated our understanding of gene expression profiles associated with biologically significant traits, including plant-microbe interactions (Kamfwa et al., 2017; Sańko-Sawczenko et al., 2019), plant responses to pathogens (Padder et al., 2016) and abiotic stress responses (Hiz et al., 2014; Recchia et al., 2018). In addition, a comprehensive high-resolution RNA-Seq atlas has been established for agriculturally important leguminous species such as soybean (Severin et al., 2010; Sreedasyam et al., 2023) and common bean (O'Rourke et al., 2014b). While significant strides have been made in common bean research utilizing RNA-Seq technology (Kamfwa et al., 2017; Recchia et al., 2018; Pereira et al., 2020; Subramani et al., 2023; Zheng et al., 2023) and the release of its genome draft to the public (Schmutz et al., 2014), the application of RNA-Seq to investigate biological nitrogen fixation (BNF) in common beans is limited. Furthermore, a comprehensive exploration of the nodule transcriptome under adverse conditions during BNF in this species has not been undertaken. Therefore, a thorough examination of how drought stress influences the nodule transcriptional profile in common beans could reveal pivotal stress-related genes affecting BNF performance, offering valuable insights into nodule functionality under challenging conditions.

In this study, we conducted an analysis of the nodule transcriptome using RNA-Seq for a highly efficient genotype for BNF, Negro Argel (Hungria and Neves, 1987), and a well-known drought-tolerant common bean genotype, BAT 477 (Beebe et al., 2013; Arruda et al., 2018), under limited water conditions. Our RNA-Seq profiling showed the up-regulation of several stress-related genes, including those associated with embryogenesis abundance (LEA), heat shock proteins (HSP), and 9-cis epoxycarotenoid dioxygenase (*NCED9*), all of which were previously identified as drought-responsive genes. Additionally, set enrichment analysis (SE) of gene ontology (GO) terms highlighted a significant down-regulation in various metabolic pathways, with a particular focus on the antioxidant metabolism within the nodules. Furthermore, we observed the regulation of specific classes of transcription factors (TFs), notably WRKY, MYB, and AP2/ERF, and several receptor-like kinases such as LyM, LRR, WAK, and LRK in response to drought stress in common bean nodules. These findings provide valuable insights into the transcriptional responses and the key pathways affected by drought stress in common bean root nodules. They contribute to a better understanding of how common bean plants adapt to growth conditions that involve BNF under drought stress.

## 2 Materials and methods

### 2.1 Plant material, growth conditions and drought-stress treatment

The two common bean genotypes Negro Argel and BAT 477 were previously selected in a greenhouse experiment in which they differ in their BNF performance when grown under symbiosis with *Rhizobium* strains and water restriction (da Silva et al., 2012a). Next, to dissect the transcriptional responses of root-nodules to drought-stress, a greenhouse experiment was carried out at Centro Nacional de Pesquisa de Agrobiologia (Embrapa - Seropédica, Rio de Janeiro State, Brazil) followed by root-nodules RNA-profiling using Illumina RNA-Seq technology. Seeds of Negro Argel and BAT 477, the former a high efficient for BNF and the latter a known drought-tolerant genotype (Hungria and Neves, 1987; Beebe et al., 2013), were surface disinfected for 5 min in 0.1% (w/v) sodium hypochlorite, sown into 1 L plastic pots containing a 2:1 (v/v) sand/vermiculite mixture and inoculated (1 mL/seed) with a mix (1:1) of the two *Rhizobium* strains broadly used as commercial inoculants in Brazil: *Rhizobium tropici* CIAT 899 (BR 322 syn SEMIA 4077), a broad host-range rhizobial strain isolated from tropical acid soils (Martínez-Romero et al., 1991); and *Rhizobium freirei* PR-F81 (syn SEMIA 4080), isolated from soil of Southern Brazil (Hungria et al., 2000), which were previously grown in yeast malt (YM) liquid medium (Fred and Waksman, 1928) over 3 days at 30°C 120 rpm.

The experiment included two water availability conditions: well-watered and drought-stress. The plants were grown in a greenhouse exposed to natural light, with daily temperatures ranging from 20-32°C and humidity fluctuating between 40% and 90%. These conditions are typical of tropical regions where common beans are commonly cultivated. Norris nutritive solution (Norris and T’mannetje, 1964) lacking nitrogen, was supplied for plant once a week until the start of drought-stress treatment. To monitor the greenhouse environment, temperature and relative humidity were measured using a Datalogger DHT-2230 device (Perceptec) (data not shown).

Throughout the experiment, we monitored soil moisture (W) using a gravimetric method for each pot, following the procedure described by Casagrande et al. (2001). The plants in the well-watered treatment were consistently kept at optimal W levels, which were approximately 80% of the field capacity, throughout the entire experiment. For the plants in the drought-stressed treatment, they were also maintained at around 80% of field capacity until 25 days after plant emergence (DAE), at which point irrigation was suspended. Well-watered (WW) and drought-stressed (DS) plants were sampled when the soil water content for the latter group reached around 5–10% to field capacity. At sampling, leaves from WW and DS plants were collected to determine the leaf relative water content (RWC) following the procedure described by Chartzoulakis et al. (1997).

For molecular analyses, nodules from WW and DS plants were separated from roots, immediately frozen in liquid nitrogen and stored at -80°C until total RNA extraction. For each treatment, a total of twelve plants from different pots were harvested and subdivided into three biological replicates, corresponding to four plants by group. Furthermore, after water deprivation a portion of 25 DAE WW and DS plants were rehydrated to a high moisture level, maintained under greenhouse conditions for 10 days (recovery period) and harvested to determined nodules’ dry weight (NDW), shoot dry weight (SDW) and shoot total nitrogen content (STNC). Some of these plants were also used to collect the whole nodulated-roots for nitrogenase activity (N2ase) analyses.

### 2.2 Evaluation of nodules’ dry weight, shoot dry weight, shoot total nitrogen content and nitrogenase activity (N2ase)

To evaluate the extent of the injury caused by the drought stress on BNF performance after recovery period, the well-watered and the rehydrated plants were harvested to assess the shoot and nodules’ dry weights (SDW and NDW, respectively), shoot total nitrogen content (STNC) determined by Kjeldahl method (Kjeldahl, 1883) and the N2ase in nodules by acetylene reduction assay (ARA) method (Vance et al., 1979). For N2ase analyses, intact nodulated roots from three plants were incubated into 10% C2H2 atmosphere for 10 minutes at room temperature and then the C_2_H_4_ content was determined by using a gas chromatography (Perkin Elmer Auto System) with flame ionization. The N2ase analyses was expressed as mmol. L^-1^. h^-1^. g. nodule^-1^.

### 2.3 Statistical analyses

The drought-stress experiment was performed in a completely randomized design and the results were presented as means with standard deviation (SD) of five independent samples by treatment (WW and DS plants) for RWC, W, SDW, NDW, STNC and N2ase analyses. Data were analyzed by Student’s t-test (p <0.05) on the Sisvar software (version 4.2), by Ferreira (2011). For RT-qPCR data, significant differences were determined by estimation of the standard error (SE) and also using the REST software version 2.0.7 (p <0.05) which performs a Pair Wise Fixed Reallocation Randomization Test (bootstrap = 2,000 permutation as default) to obtain p values (Pfaffl et al., 2002 - http://www.gene-quantification.info).

### 2.4 Root-nodules Transcriptome analysis by RNA-Seq using Illumina HiSeq 2000 platform

#### 2.4.1 RNA isolation and sample preparation

Total RNA was extracted from nodules of WW and DS plants using the Tris-SDS method (Ragueh et al., 1989). The nodules were ground to a fine powder in liquid nitrogen using a pestle and mortar, and 300 mg from those samples were used. The RNA was precipitated overnight at 4°C in 2.5 M LiCl, dissolved in 100 μL of nuclease free water and stored at -80°C until molecular analyses. The integrity of total RNA was examined by 2% (w/v) agarose gel electrophoresis looking for distinct ethidium bromide-stained rRNA bands followed by quantification using the Thermo Scientific NanoDrop ND-1000 spectrophotometer (NanoDrop Technologies, Wilmington, DE, USA). Contaminating DNA was removed from RNA using DNase I (Epicentre Biotechnologies, Madison, WI, USA) and the RNA was quantified again using Qubit® 2.0 Fluorometer (Invitrogen, Waltham, MA, USA) for more accurate quantification after DNase I treatment to ensure adequate total RNA for downstream analyses.

#### 2.4.2 cDNA library construction and RNA-Seq profiling of common bean root-nodules

To prepare the cDNA libraries for Negro Argel and BAT 477 well-watered (WW) and drought-stressed (DS) plants, the total RNA from root-nodules was processed using a TruSeqTM RNA Sample Preparation Kit according to manufacturer instructions (Illumina, San Diego, CA, United States of America). Next, these samples were submitted to high-throughput parallel sequencing using the Illumina HiSeq 2000 Platform by a commercial service provider (Fasteris, Plan-les-Ouates, Switzerland). In our analyses, a total of eight cDNA libraries, encompassing two independent biological replicates by treatment, were sequenced to generate about 153 million of 100 bp (base pair) single-end (SE) reads and perform RNA-Seq profiling.

#### 2.4.3 Mapping of short reads and assessment of differential gene expression

The FASTQ files for the eight cDNA library containing the raw sequences from the Illumina HiSeq 2000 Platform were analyzed using publicly available tools. In this case, the output data was filtered using FastQC (Andrews, 2010) and the high-quality reads obtained were trimmed using FASTX-Toolkit. Only those reads with quality above 20 (Q >20) were used. After trimming the resulting high-quality reads were aligned against the detailed *Phaseolus vulgaris* reference genome v1.0 (Schmutz et al., 2014) using a series of programs, including Bowtie v1.0 (Langmead et al., 2009) and TopHat2 v2.1 for read mapping (Kim et al., 2013). Then, the mapped reads counting was performed using HT-Seq-count version 0.5.3p9 (Anders et al., 2014). The differential expression (DE) was estimated and tested by using three different statistical algorithms: CuffDiff (Trapnell et al., 2012), DESeq1 (Anders and Huber, 2010) and edgeR (Robinson et al., 2010), a software package in R-Bioconductor 2.15. Transcripts with adjusted p-value ≤ 0.05 and estimated |log2 (FC)| ≥ 2 (up-regulated) or |log2 (FC)| ≤ 2 (down-regulated) detected by the three software were considered as differentially expressed genes (DEGs) and selected for functional annotation and downstream analyses. The RNA-Seq experiment workflow as well as the bioinformatics pipeline used in our analyses are depicted in **Supplementary Figure S1**.

#### 2.4.4 Functional annotation of differentially expressed genes

To understand the molecular responses of root-nodules to drought-stress as well as the differences between the two common bean genotypes, we submitted the common bean DEGs to BLASTn analysis (Altschul et al., 1990) against the *Phaseolus vulgaris* reference genome v1.0, available in the Phytozome v12.1 data base (Goodstein et al., 2012) and recovery the functional annotations. In addition, the genes and pathways showing the most relevant responses of common bean nodules to drought-stress were identified by set enrichment analysis (SEA) of GO terms using the AgriGO software v2.0 available at http://bioinfo.cau.edu.cn/agriGO/ (Tian et al., 2017). Based on SEA analysis, a hypergeometric test (p-values ≤ 0.005; FDR ≤ 0.05) was applied to identify which biological processes (BP) and molecular functions (MF) were overrepresented in our list of DEGs. In addition, functional analyses were performed to obtain a representative overview of the pathways affected by drought-stress in common bean nodules mapping the DEGs into *Phaseolus vulgaris* v1.0 genome (Schmutz et al., 2014) available in the MapMan software v3.6 (Thimm et al., 2004). MapMan is a user-driven tool that displays large genomic datasets onto diagrams of different metabolic pathways and biological processes. Next, based in the functional categories potentially involved in the nodule responses to drought, ten DEGs were selected for validation using reverse transcription-quantitative PCR (RT-qPCR).

### 2.5 RT-qPCR analyses for validation of RNA-Seq data

#### 2.5.1 Primer design

To validate our RNA-Seq profiling experiment, reverse transcription quantitative PCR (RT-qPCR) analyses were carried out for ten DEGs identified in common bean nodules, five up-regulated and five down-regulated. Hence, specific primers for these genes were designed using the Primer3Plus Software (Untergasser et al., 2007), and the following criteria were set out: primer length of 19-22 nucleotides; annealing temperature between 58-62°C; GC content of 50-80% and product size ranging from 100-180 base pairs. Primers’ parameters were further checked and adjusted using Oligo Explorer 1.2 software (Gene Link - http://www.genelink.com/tools/gl-oe.asp).

#### 2.5.2 First-strand cDNA synthesis

Total RNA samples from nodules of Negro Argel and BAT477 common bean genotypes, previously treated with DNase I, were used for first-strand cDNA synthesis. The presence of contaminant DNA was checked by endpoint PCR and no genomic DNA was detected in any sample tested in this work (data not shown). First-strand cDNAs syntheses were performed using 4 µg of DNase I treated total RNA, 1 µL oligo (dT) primer (1 µg/µL) and SuperScript III Reverse Transcriptase™ Kit (Invitrogen, Waltham, MA, USA) following the manufacturer’s instructions. The cDNA was diluted 1:50 in nuclease free water and then used for qPCR analyses.

#### 2.5.3 RT-qPCR experiment and data analysis

Quantitative PCR was performed on the 7500 Fast Real Timer PCR System using the SYBR Green PCR Master Mix (Applied Biosystems, Carlsbad, CA, USA). The reactions consisted of 7.5 μL of SYBR Green PCR Master Mix, 10 μM of forward and reverse primers for both target (**Table 1**) and reference genes (**Supplementary Table S1**), and 7.5 μL of 1:50 diluted fist-strand cDNA template in a total volume of 16 μL. Cycling was performed using the default conditions of the 7500 Software v2.0.5: 2 min at 95°C, followed by 40 cycles of 20 s at 95°C and 30 s at 60°C. Three technical replicates and non-template controls were used, as well as three independent biological samples for each experimental condition (biological replicates) were analyzed. Each primer sets’ efficiencies and the optimal quantification cycle threshold (Cq values) were estimated by Miner Software (Zhao and Fernald, 2005).

**Table 1:** Description of genes and primers used in the RT-qPCR and comparison of RT-qPCR and RNA-Seq experiments results.

Gene Abbreviation	*Phaseolus vulgaris* locus (Accession Number)	Gene description	Primers’ sequence (5’-3’)	Eff± SD**	qPCR	Log2FC N. Argel	Log2FC BAT 477
Up-Regulated DEGs							
*PvBdzp-Receptor*	Phvul.001G205900.v1.0 (XM_007163034.1)	Benzodiazepine receptor-related (ABA Hormone)	TCCTCTTGTGTTCGCTGTTG GGCAAGGCTTTATCAGACCA	1.886 ± 0.004	Confirmed	Non Detected on RNA-Seq	**3.7**
*PvRPK-LRR*	Phvul.005G007100.v1.0 (XM_007148637.1)	RPK-LRR-disease- resistance-protein	GGACTTGTCGTGGAACAGGT TTCATCTCGGGAAACGGGT	1.889 ± 0.007	Confirmed	Non Detected on RNA-Seq	**4.2**
*PvUSP*	Phvul.001G206900.v1.0 (XM_007163045.1)	Universal Stress Protein	AGTGGAAGTGGTGGAAGGTG TGCAGTGTGCATGGTGAGCA	1.892 ± 0.003	Confirmed	Non Detected on RNA-Seq	**3.0**
*PvMYB41*	Phvul.005G109100.v1.0 (XM_007149846.1)	MYB transcriptional factor family	AACAAGTGGTCGGCAATAGC GAGCGTGTGTAACAGGGTCA	1.894 ± 0.004	Confirmed	Non Detected on RNA-Seq	**4.2**
*PvWRKY56*	Phvul.008G058000.v1.0 (XM_007139715.1)	WRKY transcriptional factor family	GAGCAAGGGCTACGTGACTC CACCTTTCCTTTTCTCCTGGT	1.876 ± 0.004	Confirmed	Non Detected on RNA-Seq	**5.8**
Down-Regulated DEGs							
*PvRCI3*	Phvul.009G038500.v1.0 (XM_007136296.1)	Rare Cold Inducible Gene 3	GCAGCAAGAGACAGTGTGGA CATTGAAGGTTGGAGGTGGT	1.883 ± 0.007	Confirmed	Non Detected on RNA-Seq	**-3.3**
*PvPer22*	Phvul.006G129400.v1.0 (XM_007147434.1)	Peroxidase 22	TGCGACGATAGTGAGTGAGC CCAGCCAGAACTGAAGAGAC	1.939 ± 0.098	Confirmed	Non Detected on RNA-Seq	**-4.4**
*PvWRKY51*	Phvul.009G138900.v1.0 (XM_007137526.1)	WRKY transcriptional factor family	CATCGGAGAAAGCAACCTC TTGACTCACTTCTGCCTTCC	1.875 ± 0.005	Confirmed	Non Detected on RNA-Seq	**-4.7**
*PvDRFP*	Phvul.004G114700.v1.0 (XM_007152199.1)	Disease resistance family protein	GCAACTCGTTCTCCAAATCC AATGGTCCAACAGTCGGGTA	1.895 ± 0.002	Confirmed	Non Detected on RNA-Seq	**-6.5**
*PvAP2-ERF034*	Phvul.008G141000.v1.0 (XM_007140714.1)	AP2 Ethylene-responsive transcription factor ERF034	CTTCTCCTCCTCCACATCCA CTTGGTGCATGAGTTTGAGC	1.884 ± 0.005	Confirmed	Non Detected on RNA-Seq	**-3.9**

Prior to target genes’ analyses, the expression stability for three candidate reference genes (*PvEf1-Alpha*, *PvAct* and *PvIDE*) was determined. The Cq values for these genes across all sample set were converted by the qBase Software v1.3.5 (Hellemans et al., 2007) into non-normalized relative quantities, and corrected by PCR efficiency as described by Hellemans et al. (2007). These quantities were imported independently into geNorm v3.5 (Vandesompele et al., 2002) and NormFinder (Andersen et al., 2004) analysis tools, which were used according to their manuals to determine the expression stability of the candidate reference genes. These data were analyzed in both programs and the two more stable genes (*PvEF1-Apha* and *PvAct*) were used for data normalization of target genes in the qBase Software analyses.

For the relative quantification (RQ) of target genes the Cq values and PCR efficiencies estimated by Miner Software were imported into the qBase Software, which estimates the RQ using the classical 2^-ΔΔCt^ method (Livak and Schmittgen, 2001); and relative to the calibrator sample (control plant); however, it accounts for multiple stably expressed reference genes to improve normalization (Hellemans et al., 2007). Significant differences were determined by REST software version 2.0.7 (p <0.05) (Pfaffl et al., 2002) as described as previously described.

## 3 Results

### 3.1 Negro Argel and BAT 477 genotypes showed different BNF performance under drought-stress conditions

**Figure 1:**
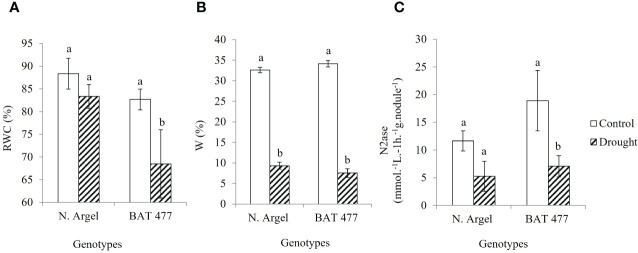
Comparison of BNF performance in Negro Argel and BAT 477 plants submitted to drought-stress. Evaluation of physiological status of common bean plants using the leaf relative water content (RWC) **(A)** and gravimetric soil moisture (*w*) **(B)**. BNF activity in drought-stressed common bean nodules estimated by acetylene reduction assay (ARA) for N. Argel and BAT 477 genotypes ten days after rehydration (recovery). Nitrogenase (N_2_ase) analyses is shown as mmol.L^-1^.h^-1^.g.nodule^-1^ **(C)**. For all analyses, different letters indicate statistical differences between well-watered and drought-stressed plants within each genotype by Fisher test (p<0.05). Standard deviation bars are shown for five biological replicates (two plants by replica).

Previously, we performed a screening experiment to select common bean genotypes which can be able to maintain their BNF capacity when submitted to drought stress. In these analyses Negro Argel and BAT 477, the former a highly efficient for BNF and the latter a known drought-tolerant genotype, respectively (Hungria and Neves, 1987; Beebe et al., 2013), showed different BNF performances when grown under water limited conditions. While Negro Argel seems to be not affected by drought, BAT 477 decreased their BNF performance in these conditions. Our findings suggested that different mechanisms to cope with drought occur in Negro Argel and BAT 477 when grown under symbiosis (unpublished data). These two genotypes were then selected to perform a new greenhouse experiment to investigate the nodule transcriptional changes in response to drought-stress. In our analyses, the shoot total nitrogen content (STNC) and shoot dry weight (SDW), which were used as phenotypic markers for BNF performance under drought stress, showed divergent patterns between the two genotypes. The STNC as well as SDW were significantly decreased in BAT 477 genotype; on the other hand, for Negro Argel these phenotypic markers remained unaffected by drought (**Table 2**). Negro Argel plants was less affected by drought (**Supplementary Figure S2**) It is noteworthy to mention that all phenotypic parameters evaluated were at least two-fold higher for this genotype when compared to BAT 477 genotype. Interestingly, despite the two genotypes being in the same gravimetric soil moisture (*w*) under stress (**Figure 1B**), BAT 477 plants began to wilt after stress treatment (**Supplementary Figure S2**) and a decline in the leaf relative water content (RWC) was observed (**Figure 1A**). Meanwhile, the Negro Argel genotype exhibited no symptoms of drought stress ten days after plant rehydration (recovery) (**Supplementary Figure S1**). Our analyses revealed a significant decrease in N2ase analyses in BAT 477, whereas its activity was only slightly affected and not statistically significant (p>0.05) in Negro Argel (**Figure 1C**). Collectively, these results confirm the distinct biological nitrogen fixation (BNF) performances of BAT 477 and Negro Argel genotypes when grown under symbiosis with Rhizobium strains and subjected to drought stress conditions, as previously observed by our group.

**Table 2:** Shoot total nitrogen content (STNC) and shoot dry weight (SDW) of common bean plants ten days after rehydration (recovery).

Genotype	Water availability regimen	STNC (mg.planta^-1^)	STNC variation (%)	SDW (g.planta^-1^)	SDW variation (%)
Negro Argel	Well-watered plants	14.87a	-13.58	0.93a	+4.3
	Drought-stressed plants	12.85a		0.97a	
BAT 477	Well-watered plants	37.13a	-34.34	1.88a	-24.47
	Drought-stressed plants	24.38b		1.42b	

The variation (%) of these parameters in drought-stressed plants related to control (well-watered plants) was estimated and the difference for BNF performance under drought conditions for Negro Argel and BAT 477 genotypes was confirmed. For all analyses, values followed by the same letter indicate that the differences were not statistically significant within each genotype by Fisher test (p<0.05). Standard deviation bars are shown for five biological replicates (two plants by replica).

### 3.2 Summary of RNA-Seq data analyses

The RNA-Sequencing output yielded about 153 million raw reads (81,499,516 for Negro Argel and 72,504,111 for BAT 477) for the eight RNS-Seq libraries. Each library represented at least 14 million reads, ranging from 14,425,608 for Negro Argel DS R1 to 29,183,581 for Negro Argel WW R1 samples (**Table 3**), a tag density sufficient for quantitative analysis of gene expression (Vargas et al., 2014; Guo et al., 2015). After stringent quality assessment and data filtering, a total of 81,489,262 and 72,497,478 high-quality reads (Q>20) were obtained for N. Argel and BAT 477 genotypes, respectively. In addition, the clean reads accounted for more than 99.98% of the total reads for both genotypes which indicates few biases in our RNA-Seq analyses (**Table 3**). These reads were then mapped to the *P. vulgaris* reference genome v1.0 (Schmutz et al., 2014) and a high number of unique mapped reads was observed for both Negro Argel and BAT 477 libraries which corresponded to about 85% of the total high-quality reads obtained.

**Table 3:** Common bean RNA-Seq experiment statistics.

Sample	Total number of reads	Reads after trimmer (Q20)	Trimmed reads	Unique aligned reads (%)
N. Argel Control R1	29,183,581	29,179,782	3,799	82.5
N. Argel Control R2	19,231,955	19,230,007	1,948	84.9
N. Argel Drought R1	14,427,803	14,425,608	2,195	83.8
N. Argel Drought R2	18,656,177	18,653,865	2,312	84.5
Total	81,499,516	81,489,262	10,254	
BAT 477 Control R1	21,104,767	21,102,985	1,782	85.5
BAT 477 Control R2	15,141,812	15,140,588	1,224	84.6
BAT 477 Drought R1	17,922,096	17,920,130	1,996	84.2
BAT 477 Drought R2	18,335,436	18,333,775	1,661	86.3
Total	72,504,111	72,497,478	6,663	

Summary of RNA-Seq data output from Illumina HiSeq 2000 platform, statistical analysis of reads number and mapping of the reads onto the common bean (*Phaseolus vulgaris* L.) reference genome.

### 3.3 Overview of the differentially expressed genes in response to drought-stress

**Figure 2:**
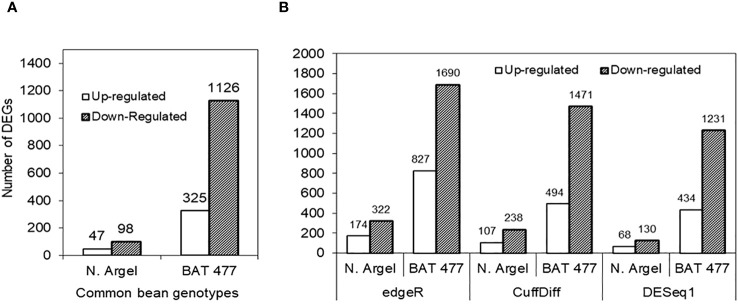
Number of differentially expressed genes (DEGs) in nodules from Negro Argel and BAT 477 common bean genotypes submitted to drought-stress. DEGs concomitantly identified by RNA-Seq in the three different statistical algorithms used for differential expression analysis: edgeR, CuffDiff and DESeq1 **(A)**, and up and down-regulated genes detected by each algorithm **(B)**.

To characterize the transcriptional responses of common bean nodules to drought, we performed RNA-profiling and the DEGs were identified by implementing three different statistical algorithms (CuffDiff, DESeq1 and edgeR). Only those genes concomitantly detected in all three algorithms were considered as responsive to drought and selected for further investigations. In our analyses, 145 and 1451 genes were differentially expressed (adjusted p-value ≤ 0.05, |log2 FC| ≥ 2.0) in Negro Argel and BAT 477, respectively. Among them, 47 (32.4%) were up-regulated whereas 98 (67.6%) were down-regulated in Negro Argel. For BAT 477 genotype, 325 genes (22.4%) were up-regulated and 1126 (77.6%) were down-regulated in nodules in response to drought. Interestingly, for both genotypes, edgeR algorithm identified the highest number of DEGs followed by CuffDiff and DESeq1. The number of DEGs identified by each algorithm as well as a pairwise comparison with the number of shared DEGs among them is summarized in **Figure 2** and **Supplementary Figure S3**.

### 3.4 Functional categorization of drought-regulated genes in common bean root-nodules

#### 3.4.1 Top 10 drought-responsive DEGs identified in each genotype

To investigate how genes are strongly regulated by drought in common root-nodules, we examined the top 10 up- and down-regulated genes (up and down-DEGs, respectively) for each genotype (**Tables 4**, **5**), as well as the shared DEGs between them (**Supplementary Figure S4**). In the case of the Negro Argel genotype, a hypothetical protein (Phvul.008G186200.v1.0) and a heat shock protein from the hsp20/alpha crystallin family (Phvul.010G155300.v1.0) were among the most strongly induced genes in nodules in response to drought (**Table 4**). Additionally, two heat shock proteins (HSP 70 and cytosolic sHSP class II), transcriptional factors (MYB and homeobox associated leucine zipper protein), and a seed maturation protein were detected among the top 10 up-DEGs. Interestingly, another hypothetical protein (Phvul_007G069800.v1.0), similar to RD29B, a protein responsive to desiccation in *Arabidopsis thaliana*, was also found in our analyses. On the other hand, genes related to glycoside hydrolase family 17 (Phvul.001G128500.v1.0) and silicon efflux transporter (Phvul.004G111900.v1.0) were the most strongly down-regulated genes in common bean nodules in response to drought. Moreover, genes belonging to important functional classes were also identified among the top 10 down-regulated DEGs, such as lipid transfer protein, cell wall associated kinase-like protein, RNA-binding and disease resistance family proteins, and a peroxidase family protein (**Table 4**).

**Table 4:** Top 10 highest and lowest expressed genes in drought-stressed common bean (*P. vulgaris* L.) nodules, Negro Argel genotype, identified in RNA-Seq experiment.

ID *P. vulgaris*	ID *M. truncatula*	E-value	Description	Log2FC
Top 10 highest expressed genes				
**Phvul.008G186200.v1.0**	Medtr5g067740.1	2E-57	hypothetical protein	5.9
**Phvul.010G155300.v1.0**	Medtr4g010320.1	1E-121	hsp20/alpha crystallin family protein	5.8
Phvul.006G116000.v1.0	Medtr8g026960.1	1E-116	homeobox associated leucine zipper protein	5.3
**Phvul.001G223700.v1.0**	Medtr7g111850.1	0.0	galactinol synthase	4.9
Phvul.003G154800.v1.0	Medtr4g130540.1	0.0	heat shock 70 kDa protein	4.9
**Phvul.009G131000.v1.0**	Medtr3g088675.1	0.0	chalcone and stilbene synthase family protein	4.6
Phvul.006G192900.v1.0	Medtr2g011660.1	1E-147	myb transcription factor	4.6
Phvul.009G080200.v1.0	Medtr3g104780.1	1E-98	cytosolic class II small heat-shock protein	4.6
Phvul.007G069800.v1.0	Medtr1g100627.1	9E-63	hypothetical protein	4.3
**Phvul.001G142000.v1.0**	Medtr7g093170.1	7E-53	seed maturation protein	4.2
Top 10 lowest expressed genes				
**Phvul.001G128500.v1.0**	Medtr4g076570.1	3E-67	glycoside hydrolase family 17 protein	-6.1
Phvul.004G111900.v1.0	Medtr6g045483.3	0	silicon efflux transporter	-5.3
Phvul.003G218900.v1.0	Medtr4g101280.1	5E-81	Lipid transfer protein	-4.2
Phvul.007G030800.v1.0	Medtr1g110180.1	0.0	wall associated kinase-like protein	-3.8
Phvul.005G140100.v1.0	Medtr4g062170.1	0.0	3-ketoacyl-CoA synthase-like protein	-3.8
Phvul.003G078000.v1.0	Medtr2g023010.1	1E-24	down-regulated protein in the presence of paraquat	-3.7
Phvul.003G278400.v1.0	Medtr8g072260.1	0.0	cytochrome P450 family ABA 8’-hydroxylase	-3.7
**Phvul.010G122000.v1.0**	Medtr7g117495.2	3E-70	RNA-binding (RRM/RBD/RNP motif) family protein	-3.6
Phvul.007G254300.v1.0	Medtr1g078490.1	0.0	disease resistance protein (CC-NBS-LRR class) family protein	-3.6
**Phvul.006G075700.v1.0**	Medtr3g467600.1	0.0	peroxidase family protein	-3.5

Common bean plants were submitted to 4 days of drought stress. Gene expression is shown as log2 fold-change (Log2FC) between control and drought-stressed plants. Bold/underlined Common bean IDs indicate common genes shared with BAT 477 genotype within the top 10 up and down expressed genes.

**Table 5:** Top 10 highest and lowest expressed genes in drought-stressed common bean (*P. vulgaris* L.) nodules, BAT 477 identified in RNA-Seq experiment.

ID *P. vulgaris*	ID *M. truncatula*	E-value	Description	Log2FC
Top 10 highest expressed genes				
Phvul.007G225200.v1.0	Medtr1g061730.1	1E-64	late embryogenesis abundant protein, putative	9.8
**Phvul.008G186200.v1.0**	Medtr5g067740.1	2E-57	hypothetical protein	8.3
Phvul.008G228100.v1.0	Medtr5g081530.1	3E-95	17.6 kDa class I heat shock protein	8.1
**Phvul.010G155300.v1.0**	Medtr4g010320.1	1E-121	hsp20/alpha crystallin family protein	8.0
**Phvul.009G131000.v1.0**	Medtr3g088675.1	0.0	chalcone and stilbene synthase family protein	7.9
**Phvul.001G223700.v1.0**	Medtr7g111850.1	0.0	galactinol synthase	7.8
Phvul.003G096700.v1.0	Medtr4g120040.1	4E-40	late embryogenesis abundant protein	7.8
Phvul.002G231400.v1.0	Medtr6g061850.1	1E-137	17.6 kDa class I heat shock protein	7.3
**Phvul.001G142000.v1.0**	Medtr7g093170.1	7E-53	seed maturation protein	7.3
Phvul.007G113700.v1.0	Medtr1g076910.1	1E-130	22.0 kDa class IV heat shock protein	7.3
Top 10 lowest expressed genes				
**Phvul.001G128500.v1.0**	Medtr4g076570.1	3E-67	glycoside hydrolase family 17 protein	-9.5
Phvul.008G086800.v1.0	Medtr7g072510.1	0.0	class III peroxidase	-8.86
Phvul.003G169100.v1.0	Medtr4g115360.2	7E-69	lipid transfer protein	-8.54
**Phvul.006G075700.v1.0**	Medtr3g467600.1	0.0	peroxidase family protein	-8.29
Phvul.011G105900.v1.0	Medtr4g046713.1	0.0	peroxidase family protein	-8.17
Phvul.003G218800.v1.0	Medtr4g101330.1	4E-75	Lipid transfer protein	-7.89
Phvul.001G050300.v1.0	Medtr1g012710.1	5E-71	protease inhibitor/seed storage/LTP family protein	-7.89
Phvul.011G047400.v1.0	Medtr4g069170.1	4E-28	lipid transfer protein	-7.79
**Phvul.010G122000.v1.0**	Medtr7g117495.2	3E-70	RNA-binding (RRM/RBD/RNP motif) family protein	-7.64
Phvul.005G111700.v1.0	Medtr2g089120.1	0.0	sesquiterpene synthase	-7.6

Common bean plants were submitted to 4 days of drought stress. Gene expression is shown as log2 fold-change (Log2FC) between control and drought-stressed plants. Bold/underlined Common bean IDs indicate common genes shared with BAT 477 genotype within the top 10 up and down expressed genes.

In our analyses, a late embryogenesis abundant (LEA) protein (Phvul.007G225200.v1.0) was the most highly expressed DEG in BAT 477 nodules (**Table 5**). Moreover, another LEA protein, genes related to HSPs, seed maturation, two synthases (galactinol synthase, chalcone and stilbene synthase), and a caa3_CtaG domain-containing hypothetical protein were found among the top 10 up-DEGs. As for the down-regulated genes in BAT 477, we found a glycoside hydrolase family 17 protein (Phvul.001G128500.v1.0) as the most strongly down-regulated gene in nodules. Additionally, similar to the Negro Argel genotype, three peroxidases and three lipid transfer proteins were down-regulated in BAT 477. Moreover, a protease inhibitor/seed storage/LTP family protein, a gene related to sesquiterpene synthase, and an RNA-binding family protein were also down-regulated (**Tables 4**, **5**).

To compare the molecular responses of Negro Argel and BAT 477 nodules under drought, we determined the common shared up- and down-regulated DEGs between these two genotypes based on the top 10 up- and down-regulated gene lists (**Supplementary Figure S4**). We found five common up-regulated genes: caa3_CtaG domain-containing hypothetical protein (Phvul.008G186200.v1.0), hsp20/alpha crystallin family protein (Phvul.010G155300.v1.0), chalcone and stilbene synthase family protein (Phvul.009G131000.v1.0), galactinol synthase (Phvul.001G223700.v1.0), and seed maturation protein (Phvul.001G142000.v1.0). There were also three common down-regulated genes: glycoside hydrolase family 17 protein (Phvul.001G128500.v1.0), peroxidase family protein (Phvul.006G075700.v1.0), and RNA-binding (RRM/RBD/RNP motif) family protein (Phvul.010G122000.v1.0). Interestingly, in both cases, the Log2FC in response to drought was more pronounced in BAT 477 nodules when compared to the Negro Argel genotype, showing higher values for |Log2FC| (**Supplementary Figure S4**).

#### 3.4.2 AgriGO search for enrichment analysis

**Figure 3:**
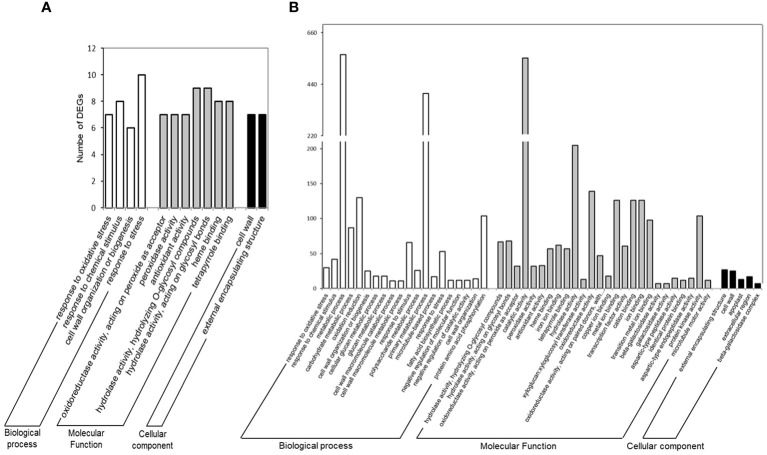
Functional annotation of differentially expressed genes (DEGs) in Negro Argel **(A)** and BAT 477 **(B)** drought-stressed nodules. Gene ontology (GO) categorization was performed after SEA analysis using AgriGO Software v2.0. Results are summarized for the three main GO categories: Biological Process, Molecular Function, and Cellular Component.

In addition to BLASTn functional annotation, a set enrichment analysis (SEA) was performed using the AgriGO software to determine which genes and pathways were the most relevant responses of common bean nodules to drought stress, and an overview of the main pathways affected during these responses, summarized for the three main gene ontology categories: “Biological Process”, “Cellular Component” and “Molecular Function”, are shown in **Figure 3**. In the “Biological Process (BP)” category, the SEA results showed significant enrichment for BAT 477 up-regulated DEGs (up-DEGs) associated with the “lipid metabolic process” (GO:0006629). This enrichment included specific terms such as “fatty acid metabolic process” (GO:0006631) and “fatty acid biosynthetic process” (GO:0006633) as significant GO terms (**Supplementary Figure S5**). However, there was no statistical significance found for Negro Argel genotype up-DEGs. Furthermore, we evaluated the down-regulated DEGs (down-DEGs), which accounted for 77.6% and 67.6% of BAT 477 and Negro Argel DEGs, respectively (**Figure 2**). Both genotypes showed enrichment in GO terms such as “cell wall organization or biogenesis” (GO:0071554) and “response to chemical stimulus” (GO:0042221). Notably, the “response to oxidative stress” (GO:0042221) term was highly significant for Negro Argel. Additionally, BAT 477 had additional enriched GO terms, including “carbohydrate metabolic process” (GO:0005975), “oxidation reduction” (GO:0055114), “phosphorus metabolic process” (GO:0006793), and “macromolecule modification” (GO:0043412). These results suggest a higher metabolic activity in BAT 477 nodules when compared to the Negro Argel genotype. For a detailed overview of the more significant GO terms in the BP category found in SEA analyses for BAT 477 and Negro Argel DEGs, refer to **Supplementary Figures S6** and **S7**, respectively.

In the “Molecular Function” (MF) category, the up-regulated DEGs were primarily associated with “transferase activity” (GO:0016746) in BAT 477 genotype (**Supplementary Figure S8**). However, for the Negro Argel genotype, similar to the BP category, no significant GO terms were found. On the other hand, when analyzing the down-regulated DEGs, significant GO terms were enriched for both genotypes. These included the terms “antioxidant activity” (GO:0016209), which encompasses “peroxidase activity” (GO:0004601), “hydrolase activity” (GO:0016787), and “heme binding” (GO:0020037) in AgriGO SEA analyses (**Supplementary Figures S9A** and **S9B** for BAT 477 and Negro Argel, respectively). Furthermore, additional GO terms specifically enriched in BAT 477 included “protein kinase activity” (GO:0004672) and “transcription factor activity” (GO:0003700). These terms include genes involved in cell signaling and gene expression regulation (**Supplementary Figure S9A**). These findings highlight the differential responses of these two genotypes to drought stress in terms of molecular functions.

#### 3.4.3 Main metabolic pathways modulated by drought-stress in nodules

In addition to the AgriGO SEA analysis, we performed an analysis using MapMan Software to gain a comprehensive overview of the main pathways affected in nodules under drought stress. Similar to the AgriGO SEA analysis, we found that DEGs from different metabolic pathways were affected in drought-stressed nodules for both genotypes (**Supplementary Figures S10**, **S11**). These pathways included those related to heat shock proteins and abiotic stress responses, which are directly involved in responding to abiotic stress. Furthermore, we observed dynamic regulation in pathways associated with cell wall metabolism, cell signaling, and transcription factor activities, among other effects.

**Figure 4:**
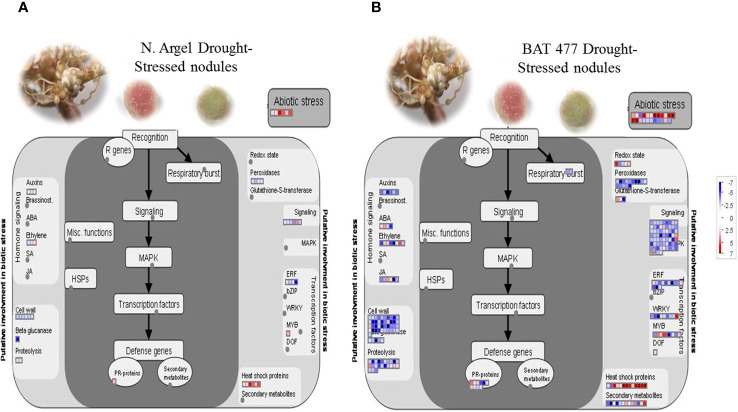
MapMan “biotic stress” overview of differentially expressed genes (DEGs) in root nodules in response to drought stress. Results for Negro Argel **(A)** and BAT 477 **(B)** genotypes inoculated with *Rhizobium tropici* BR520 and BR534 strains and submitted to four days of drought stress are shown. Blue stands for down-regulated and red stands for up-regulated genes. Only DEGs are shown, with adjusted p-value ≤ 0.05, |log2FC| ≥ 2.0 differences from control.

For a more detailed perspective, we used the MapMan “biotic stress” (**Figure 4**) and “regulation overview” (**Supplementary Figures S10**, **S11**) files for mapping the DEGs. Our results indicated that abiotic stress-related genes were induced in Negro Argel and BAT 477 genotypes. Furthermore, both genotypes exhibited several up-regulated DEGs in the “heat shock proteins” and “abiotic stress” categories Interestingly, only down-regulated DEGs were mapped to “peroxidases” within the antioxidant metabolism-related category and “auxins” in the hormone signaling and metabolism pathway. A great number of DEGs in hormone signaling and metabolism, cell wall metabolism, cell signaling, transcription factors (TFs), and proteolysis (in the case of BAT 477) were down-regulated. Additionally, PR-proteins, which play a role in plant-pathogen interactions, displayed dynamic regulation in BAT 477 drought-stressed nodules, with some DEGs being up-regulated and others down-regulated (**Figure 4B**), whereas only one PR-protein was mapped to this category in Negro Argel (**Figure 4A**).

To gain insight into the intricate regulation of common bean nodule responses to drought stress, we utilized the “regulation overview” map file in MapMan functional analyses. While some DEGs in categories like TFs and receptor kinases were up-regulated in drought-stressed common bean nodules (**Supplementary Figures S10**, **S11**), the prevailing trend for both BAT 477 and Negro Argel genotypes was down-regulation across several processes. Transcriptional repression was observed in genes related to receptor kinases, G-proteins, calcium signaling, transcription factors, protein modification and degradation, the ascorbate-glutathione cycle, and genes involved in regulation mediated by the phytohormones auxin, gibberellic acid, and jasmonate. Interestingly, these responses were more pronounced in BAT 477, and in certain instances, some processes were observed exclusively in this genotype, such as the regulation pathways related to ABA, jasmonate, and gibberellic acid (**Supplementary Figures S10**, **S11**).

For a more focused discussion, we emphasize on key gene categories potentially involved in plant responses to abiotic stresses based on the AgriGO SEA and MapMan analyses, as well as processes related to nodule responses under drought stress. We examined both down- and up-regulated DEGs in the following categories: “heat shock proteins,” “cell signaling,” “transcription factors,” “hormone metabolism and signaling,” and “antioxidant activity.” These selected categories, with the exception of hormone metabolism, showed significant enrichment in our AgriGO SEA analysis (see AgriGO results).

#### 3.4.5 Heat shock proteins and abiotic stress responses

In our MapMan analysis, we identified thirteen DEGs within the “heat shock proteins” (HSPs) category for BAT 477 and five DEGs for the Negro Argel genotype (**Figure 4**; **Supplementary Tables S2, S3**). Additionally, some genes related to the “abiotic stress responses” category, which includes HSPs, were found in BAT 477 DEGs. Unlike HSPs, DEGs related to cold, drought, salt, and light responses were generally down-regulated in nodules in response to drought in BAT 477. Notably, a gene related to cold stress, COLD-REGULATED 413-PLASMA MEMBRANE 2 (COR413-PM2), was up-regulated in BAT 477 nodules, indicating potential crosstalk between cold and drought stress responses (**Supplementary Table S2**). We also identified a universal stress protein (USP) in the BAT 477 DEGs.

#### 3.4.6 Cell signaling

We identified DEGs related to cell signaling. In the case of BAT 477, we found sixty-nine DEGs that were mapped to this category. These included three genes involved in signaling related to sugar and nutrient physiology, 41 receptor kinases of LRR and DUF26 types, along with other kinases. Additionally, there were eleven genes associated with calcium signaling and eight DEGs related to G-protein signaling, among other signaling processes (**Supplementary Table S2**). Notably, many of the DEGs in this category showed down-regulation in their Log2FC values. In contrast, for Negro Argel, only five DEGs related to receptor kinases and one DEG involved in calcium signaling were identified (**Supplementary Table S3**). Once again, a significant proportion of these DEGs in the signaling category exhibited down-regulation.

#### 3.4.7 Transcription factors

Transcriptional regulation plays a crucial role in plant responses to environmental constraints. In our analysis, we observed the regulation of several classes of transcription factors (TFs) in response to drought stress in both BAT 477 and Negro Argel genotypes (**Supplementary Tables S2, S3**). For BAT 477, the major classes of TFs regulated by drought included MYB, WRKY, and AP2-EREBP (APETALA2/Ethylene-responsive element binding protein) TFs. Notably, a majority of the WRKY and AP2-EREBP genes were down-regulated, while among the nine MYB TFs, five were up-regulated in response to drought. In the case of Negro Argel, we identified five TFs that were mapped (**Supplementary Table S3**). Among these, one MYB TF was up-regulated, while four AP2-EREBP (APETALA2/Ethylene-responsive element binding protein) TFs were down-regulated in nodules in response to drought.

#### 3.4.8 Hormone metabolism and signaling

While our AgriGO analyses did not reveal gene ontology terms related to hormones, we identified several DEGs associated with hormone metabolism and signaling in both the “biotic stress” and “regulation overview” MapMan files (**Supplementary Tables S2, S3**). For BAT 477, we found twenty-four genes linked to abscisic acid (ABA), auxin, ethylene, and jasmonate phytohormones that were regulated by drought. Among these DEGs, only five genes were up-regulated: two genes regulated by ABA, one involved in ethylene synthesis/degradation, and two involved in jasmonate synthesis/degradation. The majority of DEGs related to hormone metabolism and signaling were down-regulated. In the case of Negro Argel, we identified three down-regulated genes related to auxin metabolism and signaling, and two genes associated with ethylene metabolism. Additionally, one gene activated by ethylene (MBF1C-MULTIPROTEIN BRIDGING FACTOR 1C) was up-regulated in response to drought stress in nodules.

#### 3.4.9 Antioxidant activity

Regarding antioxidant metabolism in nodules under drought stress, we observed a significant decrease in the expression of peroxidase genes for both BAT 477 and Negro Argel genotypes (**Supplementary Tables S2, S3**). Additionally, for BAT 477, the regulation of other classes of genes related to antioxidant metabolism, including glutathione S-transferase, thioredoxin, ascorbate-glutathione, and glutaredoxin, was affected. Interestingly, two DEGs associated with the ascorbate-glutathione cycle were down-regulated, indicating a reduction in antioxidant protection at the transcriptional level.

### 3.5 Validation of transcriptome data and evaluation of the expression profile of genes related to drought stress responses in common bean nodules

**Figure 5:**
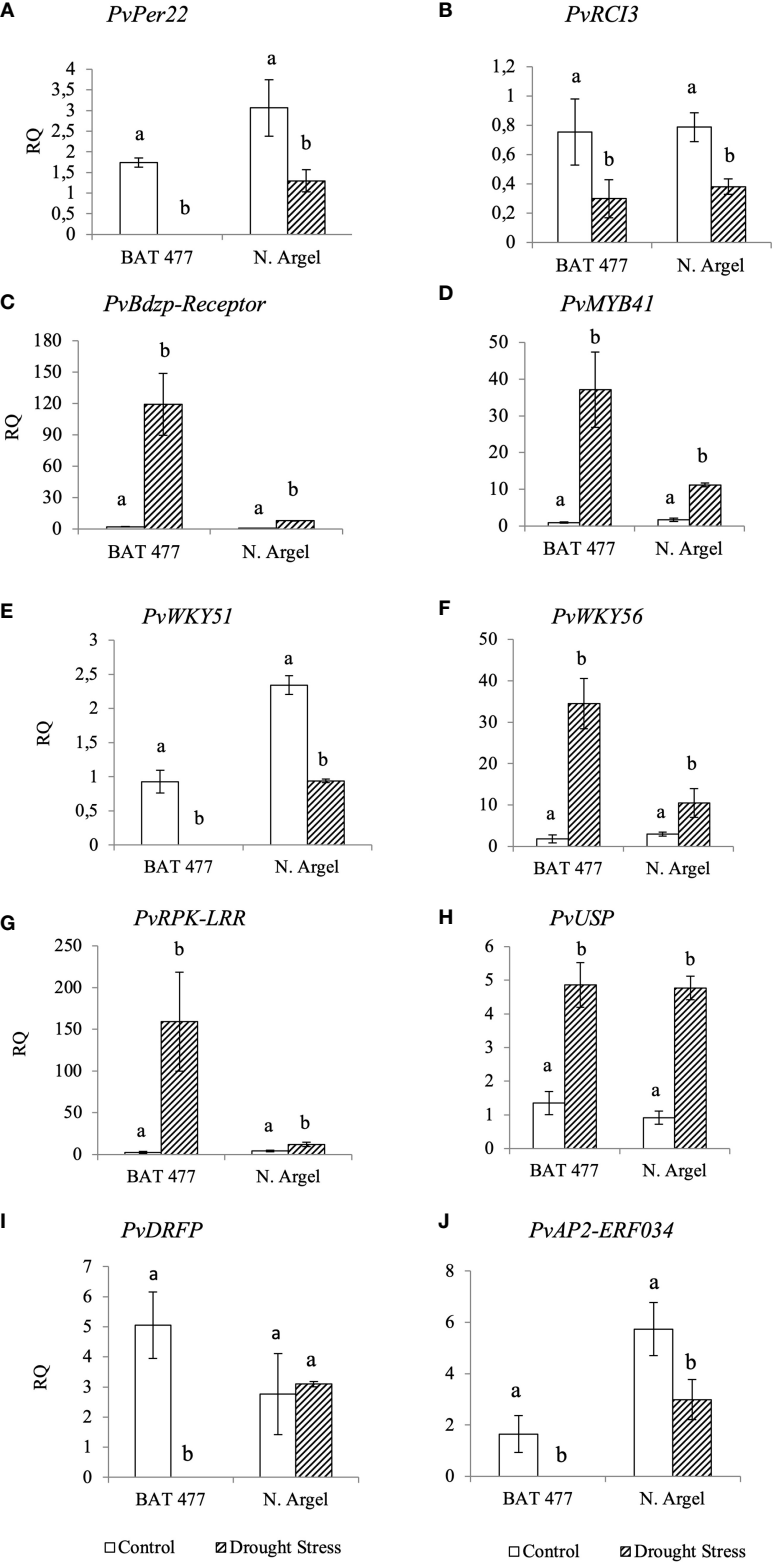
Expression profiles of differentially expressed genes (DEGs) by RT-PCR for RNA-Seq transcriptome validation. The expression profile of ten DEGs identified in RNA-Seq analyses was confirmed by RT-qPCR using cDNA from BAT 477 and Negro Argel control and drought-stressed nodules. The reference genes *PvEf1-Aplha* and *PvAct* were previously evaluated on geNorm and NormFinder algorithms (**Supplementary Figure S7**) and used for data normalization. For the genes *PvPer22*, *PvWRKY51*, *PvDRFP* and *PvAP2-ERF034*, the Cqs were not detected for “BAT 477 drought stress” samples. **(A)** *Per22*: Peroxidase 22; **(B)** *RCI3*: Rare Cold Inducible 3 gene; **(C)** *PvBdzp-Receptor*: Benzodiazepine receptor-related; **(D)** *PvMYB41*; **(E)** *PvWRKY51* and *PvWKY56* **(F)**: *WRKY* transcription factors, **(G)** *PvRPK-LRR:* RPK-LRR-disease*-* resistance-protein, **(H)** Universal stress protein; **(I)** *PvDRFP:* Disease resistance family protein, **(J)** *PvAP2-ERF034:* AP2 Ethylene-responsive transcription factor ERF034. Standard deviation (SD) bars are shown for biological replicates. Values followed by different letters indicate significant differences (p<0.05) between control and drought-stressed nodules by genotype as estimated by REST Software analysis. RQ, Relative quantification.

**Figure 6:**
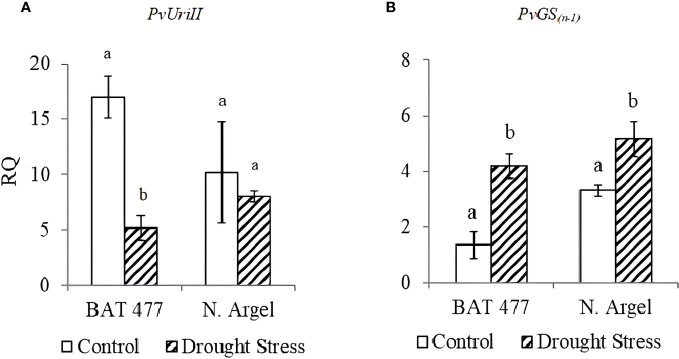
Expression profiles of two genes related to nitrogen metabolism and assimilation in common bean nodules upon aging senescence. *PvUriII*: Uricase II, involved in ureides biosynthetic pathway **(A)**; *PvGS(n-1):* Glutamine synthetase gamma-subunit, an enzyme responsible for the assimilation of NH_4_^+^ into organic nitrogen **(B)**; Standard deviation (SD) bars are shown for biological replicates. Values followed by different letters indicate significant differences between control and drought-stressed nodules by genotype as estimated by the REST software version 2.0.7 (p<0.05). RQ, Relative quantification.

To validate our RNA-Seq results, we selected 10 DEGs (5 up-regulated and 5 down-regulated) based on the main pathways affected by drought stress as identified in the AgriGO analysis (see **Figure 3** and **Supplementary Figures S5**–**S9**) and MapMan analysis (**Figure 4**; **Supplementary Figures S10–S11**). Before conducting RT-qPCR analysis of these DEGs, we assessed the expression stability of three candidate reference genes (*PvEf1-Alpha*, *PvAct*, and *PvIDE*). This was done using two different algorithms, geNorm and NormFinder Software. Both algorithms identified *PvEf1-Alpha* and *PvAct* as the most suitable reference genes for normalizing the qPCR data under our experimental conditions (**Supplementary Table S1**; **Supplementary Figure S12**). In the RT-qPCR experiments (**Table 1**), we observed that *PvPer22*, *PvRCI3*, *PvWRKY51*, and *PvAp2-ERF034* were down-regulated in both genotypes, whereas *PvDRFP* was down-regulated only in the BAT 477 genotype. On the other hand, *PvBdzp*-Receptor, *PvMYB41*, *PvWRKY56*, *PvRPK-LRR*, and PvUSP were up-regulated in response to drought in both genotypes (**Figure 5**). Notably, in the BAT 477 drought stress samples, Cq was not detected for the genes *PvPer22*, *PvWRKY51*, *PvDRFP*, and *PvAp2-ERF034*. The expression profiles of genes related to nitrogen metabolism and assimilation in common bean nodules were also different for the two genotypes under stress. PvUriII, involved in the ureide biosynthetic pathway, was severely down-regulated in BAT 477 and almost unaffected in N. Argel. On the other hand, PvGS(n-1), an enzyme responsible for the assimilation of NH4+ into organic nitrogen, was significantly induced under stress for both genotypes (**Figure 6**). Furthermore, similar to the RNA-Seq transcriptome analysis, the relative quantification (RQ) values were higher for BAT 477 (up-regulated DEGs) or lower (down-regulated DEGs) compared to Negro Argel. This indicates a significant impact of drought stress in the BAT 477 genotype. Overall, these results are consistent with the RNA-Seq analysis, providing strong validation for our nodule transcriptome data, confirming the up- and down-regulated genes in both genotypes.

## 4 Discussion

The development of next-generation sequencing (NGS) technology has provided unprecedented opportunity and a powerful tool for gene expression profiling in plants (O'Rourke et al., 2014a; Schmutz et al., 2014). To our knowledge, previous studies using RNA-Seq technology concerning to BNF in common bean are scarce (O'Rourke et al., 2014b; Kamfwa et al., 2017) mainly those focused on transcriptional responses in root-nodules tissue. Hence, in the present study, we used the Illumina HiSeq 2000 platform to examine BNF under abiotic stress conditions. We focused on understanding the transcriptional changes in nodules from two common bean genotypes in response to drought. For this purpose, the plants were inoculated with a mixture of *Rhizobium tropici* strains and submitted to four days of drought stress by suspension of irrigation.

In our conditions, the BNF activity was severely affected by drought in BAT 477 genotype showing a significant decreasing in N2ase analyses as well as STNC and SDW, which were evaluated ten days after plants rehydration (**Figure 1C** and **Table 2**). On the other hand, Negro Argel was not significantly affected in any of these parameters which suggest a lower susceptibility to drought during symbiosis in this genotype. Hence, the STNC, SDW and N2ase analyses phenotypic markers allowed evaluate the BNF performance of the two genotypes after stress and the contrasting susceptibility of Negro Argel (tolerant) and BAT 477 (susceptible) to drought stress under BNF conditions was confirmed. Summerfield et al. (1985) observed that the drought-tolerant cultivar, such as Negro Argel, showed greater maintenance of leaf area under water deficit, resulting in greater productivity. This trait can be important for BNF drought tolerant as well. Pimentel and Cruz Perez (2000), in order to establish parameters for drought tolerance evaluation in common bean genotypes showed that the SDW, along with leaf water potential (Ψf) and leaf area, is a suitable marker of the effect of drought. Interestingly, although BAT 477 has been considered a drought-tolerant genotype under N fertilized field conditions (Sponchiado et al., 1989; Guimarães, 1996), in our conditions BAT 477 plants began to wilt after drought with a 68.5% RWC value (**Figure 1A**). On the other hand, Negro Argel genotype showed no symptoms of drought stress (**Supplementary Figure 1**), showing the RWC unaltered (**Figure 1A**), despite the low value of substrate humidity measured by gravimetric soil moisture (w) was very similar for both genotypes (**Figure 1B**). Similar results have been reported by Gomes et al., 2000 in which Negro Argel genotype presented lower reductions in stem biomass at the end of growth cycle, in leaf area duration, and in grain yield, demonstrating a higher drought tolerance. Taken together, these results suggest that Negro Argel drought tolerance under BFN conditions differ from BAT 477 genotype by using a different mechanism to overcome the drought even under similar conditions of irrigation.

The drought tolerance observed in BAT 477 under field conditions is mainly attributed to its extensive and deep root system when growing in soils with insufficient water or nutrient supplies (Sponchiado et al., 1989). However, in our experimental setup, the effectiveness of this mechanism is constrained by the substrate volume explored by the root system. Some studies have suggested that common beans growing under conditions of nitrogen (N_2_) fixation exhibit greater drought tolerance than those supplied with sufficient levels of nitrogen fertilizer (NH_4_NO_3_) (Lodeiro et al., 2000). Nevertheless, the understanding of the effects of BNF on drought tolerance in common beans remains unclear and depends on various factors, including genotype (Ramos et al., 2003; Devi et al., 2013) and the specific symbiotic combination (Tajini et al., 2012; Hungria and Kaschuk, 2013).

In the present study, an RNA-Seq nodule transcriptome analysis was carried out using Illumina High-Seq 2000 platform. Comparative transcriptional level analysis of drought-stressed and control plants using three different statistical algorithms (CuffDiff, DESeq1 and edgeR) revealed a higher number of down-regulated DEGs in susceptible genotype (BAT 477) when compared to the drought tolerant one (Negro Argel) (**Figure 2**; **Supplementary Figure S3**). Overall, there was much more genes responsive to drought in BAT 477 suggesting a pronounced effect of drought stress for this genotype. In agreement with our results, a higher number of DEGs as well as down-regulated genes was observed for a drought-sensitive genotype of common bean (Wu et al., 2014). Moreover, in a salt tolerant common bean genotype a great number of down-regulated genes in roots and leaves submitted to salt stress was found (Hiz et al., 2014). Although a remarkable difference in DEGs number among the two contrasting genotypes has been observed, some common up- and down-regulated genes were found within the top 10 DEGs including one small heat shock protein (up-regulated) and one peroxidase family protein (down-regulated) (**Supplementary Figure S4**) suggesting the recruitment of shared pathways in drought stress responses.

Throughout BNF, nodule oxygen (O_2_) permeability regulated by the O_2_ diffusion barrier (ODB) is among the main processes limiting nodule performance (Arrese-Igor et al., 2011). Under drought stress, a decline in the permeability to O_2_ diffusion leading to a reduction in nodule respiration and therefore a lower production of energy via ATP synthase has been described (Serraj and Sinclair, 1996). Interestingly, in our analysis a hypothetical protein containing cytochrome c oxidase assembly factor CtaG domain (GeneBank: XP_007141322.1) was highly induced in both genotypes (**Supplementary Figure 2**). Since BNF is an energy demanding process, a high rate of oxygen respiration is necessary to supply this requirement. Cytochrome C oxidase shows high affinity to O_2_, and its induction in nodules may be contributing to maintain respiration rate at low O_2_ levels and consequently nodule performance under drought stress.

Several BAT 477-Negro Argel shared up-regulated genes were identified in the top 10 DEGs. Among them, galactinol synthase (GenBank: XP_007163306.1) plays a crucial role in the biosynthesis of raffinose family oligosaccharides (RFOs), functioning as osmoprotectants with potential stress mitigation properties (Santos et al., 2015). Additionally, a chalcone/stilbene synthase (CSH) family protein (GenBank: XP_007137487.1) was found, which serves as a key enzyme in the flavonoid/isoflavonoid biosynthesis pathway. This enzyme is induced in plants under various stress conditions such as UV light exposure and bacterial or fungal infections. The expression of CHS leads to the accumulation of flavonoids and isoflavonoids, contributing to the plant’s defense mechanism, particularly through the salicylic acid defense pathway (Dao et al., 2011).

Drought tolerance is a complex trait that involves several molecular, biochemical, and physiological mechanisms to avoid or tolerate periods of drought stress (Todaka et al., 2015). The MapMan analysis of DEGs provided a comprehensive overview of the key pathways influenced by drought stress in nodules (**Figure 4** and **Supplementary Figures S10** and **S11**), aligning with the enriched Gene Ontology (GO) terms identified in AgriGO SEA (**Supplementary Figures S8** and **S9**). Collectively, our results imply comparable responses to drought stress between the two genotypes. Nonetheless, BAT 477 exhibited a higher number of genes in all drought-affected pathways (**Figure 4B**), likely linked to its heightened susceptibility to drought, as evidenced in analyses of shoot dry weight (SDW), total nitrogen content (STNC), and nitrogenase activity (N2ase) (**Figure 1**). Furthermore, BAT 477 displayed exclusive regulation of certain pathways, including “ABA and jasmonate hormone signaling,” “redox state,” “glutathione-S-transferase,” and “secondary metabolism”.

As sessile organisms, cell signaling and transcriptional regulation of gene expression is a crucial trait in plant responses to environmental constraints (Li et al., 2014). Moreover, signal-transduction genes are important at the different stages of *Rhizobium* and leguminous symbiosis as well as the maintenance of nodule metabolism during BNF (Carvalho et al., 2013). In our analysis, several signaling-related genes were responsive to drought in nodules for the two genotypes (**Supplementary Tables S2, S3**). These DEGs included signaling genes related to sugar and nutrient physiology processes, receptors kinases such as LRR and DUF 26 types, G proteins and calcium signaling transduction related-genes. Plant membrane receptors and sensor proteins play important roles in various signaling pathways, conveying information to their cytoplasmic target proteins via catalytic processes, such as phosphorylation. Moreover, it has been hypothesized that these signaling proteins are involved in the initial process of water status perception outside the cell which includes calcium signaling and receptors-like kinases (Maathuis, 2014). In Arabidopsis, the RLK receptors family includes more than 600 members, with the leucine rich-repeat (LRR)-RLKs constituting the largest subgroup (Gish and Clark, 2011). Interestingly, for Negro Argel genotype the number of genes in this category was very limited when compared to BAT 477 (**Supplementary Tables S2, S3**). Moreover, the majority of signaling genes were down-regulated in both genotypes and a further characterization of them, and the integrated investigation of the changes in nodule metabolism mediated by differential gene expression, could give insights on the signal transduction pathways to reprogramming nodule metabolism in response to drought.

Several transcription factors play important roles in translating stress signals into changes in gene expression (Li et al., 2014). In our analysis, some classes of TFs were regulated by drought in both genotypes (**Supplementary Tables S2, S3**) and the major TFs identified were MYB, WRKY and AP2-EREBP/AP2-ERF. These TFs have been reported to be involved in abiotic stress responses in different species (Oh et al., 2011; Niu et al., 2012; Wu et al., 2014). Similar results were found by Hiz et al. (2014) which used RNA-Seq approach to investigate the transcriptional responses to salt stress in leaves and roots of common bean plants in which AP2-EREBP, WRKY and MYB were identified among the most abundant differentially expressed TF families. Recently, WRKY and MYB genes were overrepresented in the common bean transcriptome analysis for drought-responsive genes discovery (Wu et al., 2014). As one of the largest TF groups in plants, the MYB family has been shown to be essential for the responses to abiotic stresses (Chen et al., 2014; Li et al., 2014). In *Arabidopsis* MYB60, a regulator of stomata movement was down-regulated by drought stress, and its overexpression resulted in hypersensitivity to water deficit (Oh et al., 2011). On the other hand, the biosynthesis of cuticular wax activated by MYB96 is required for the drought tolerance of plants (Seo et al., 2009; Seo et al., 2011) and the gene AtMYB41 is induced by drought and may function as a transcription factor in modulating cell expansion and cuticle deposition during drought stress (Cominelli et al., 2008). These results showed the dynamic regulation of this TF family on drought stress responses in plants. Additionally, other two important TF families were found in our DEGs. The AP2/ERF family proteins are plant-specific transcription factors involved in plant abiotic stress responses (Mizoi et al., 2012) and the majority of these TFs was down-regulated in our data set. Similar to AP2/ERF genes, WRKY-type transcription factors are involved in multiple aspects of plant growth, development, and stress responses (Chen et al., 2012). Moreover, a single WRKY gene can be simultaneously regulated by several stress factors (Wei et al., 2008; Niu et al., 2012), showing its diverse regulatory function on transcriptional reprograming during plant stress responses. In wheat, the genes *TaWRKY2* was regulated by drought, salt, and ABA meanwhile *TaWRKY19* was regulated by cold, drought, salt, and ABA treatments (Niu et al., 2012). *TcWRKY53* is also simultaneously induced by cold, salt and PEG treatments (Wei et al., 2008). Some works have reported WRKY proteins as key components of ABA (Shang et al., 2010) and ROS signaling (Davletova et al., 2005), which are among the major events triggered in the plant cells when submitted to stresses (Cruz de Carvalho, 2008). In *Arabidopsis*, the heterologous expression of two wheat WRKY genes (*TaWRKY2* and *TaWRKY19)* conferred tolerance to multiples abiotic stresses by up-regulation of downstream genes responsive to stress (Niu et al., 2012).

In addition to signal transduction and transcription factors related-genes, we found in our DEGs some transcripts involved in plant hormone signaling and metabolism, such as abscisic acid, auxin, ethylene and jasmonate (**Supplementary Tables S2, S3**). Ethylene is known as a senescence inducer and stress-related phytohormone. In agreement with this assumption, in our analysis the gene 1-aminocyclopropane-1-carboxylate oxidase (Phvul.008G214200.1), which is involved in the key step of ethylene biosynthetic pathway, was up-regulated in nodules of BAT 477, but not in Negro Argel genotype, in response to drought. On the other hand, the majority of genes, involved in ethylene signaling and metabolism as well as other phytohormones, were down-regulated and this response in nodules needs further investigation.

Some environmental conditions, such as drought or salinity, are responsible for nodule senescence and also cause an O_2_ content imbalance, which is necessary to ensure a successful BNF activity (Puppo et al., 2005). Moreover, the oxidative stress in plants negatively affects the cell membrane, nucleic acids, and proteins structures, leading to metabolic disturbances (Cruz de Carvalho, 2008). Although some works have described an increase in antioxidant responses under drought (Naya et al., 2007). A remarkable down-regulation in antioxidant metabolism related-genes was observed, mainly for BAT 477 genotype (**Supplementary Tables S2, S3**; **Figure 6**) and included gene related to ascorbate-glutathione cycle, which is the major antioxidant cytosolic mechanism operating in the nodule (Becana et al., 2010), suggesting a decreasing on antioxidant protection at transcriptional level. In agreement with these findings, some studies reported that severe drought stress decreased antioxidant activity in nodules (Gogorcena et al., 1995; Porcel et al., 2003), which could partially explain the results observed for BAT 477, the susceptible genotype.

Heat shock proteins (HSPs) play a fundamental role in protecting plants against stress by re-establishing normal protein conformation and thus cellular homeostasis (Wang et al., 2004). In our results, the induction of HSP-encoding genes suggests an acclimation response of nodules to maintain its metabolism (**Figure 4**; **Supplementary Tables S2, S3**). In cowpea, a high tolerant leguminous species, the induction of HSPs has been reported in nodules under drought and heat stresses (Simoes-Araujo et al., 2008; da Silva et al., 2012b). Moreover, the induction of HSPs was correlated to heat tolerance in different genotypes of common bean (Simões-Araújo et al., 2003). Interestingly, a COLD-REGULATED 413-PLASMA MEMBRANE 2 gene (COR413-PM2) was up-regulated in BAT 477 nodules suggesting a crosstalk among cold and drought responses (**Figure 6B**; **Supplementary Table S2**). The COR413-PM2 is a stress-regulated multispanning transmembrane family protein restrict to the plant kingdom which is correlated with the development of freezing tolerance in cereals and Arabidopsis and several members of those family are also regulated by drought stress, light, and abscisic acid (Breton et al., 2003).

In this study, 10 DEGs were selected for RNA-Seq validation by RT-qPCR and our results showed that the expression profile of DEGs was very similar to RNA-Seq results (**Figure 5**). In some cases, such as *PvPer22*, *PvWRKY51*, *DRFP* and *PvAP2-ERF034*, the transcripts abundance of these genes was undetectable for BAT 477 drought stressed samples which suggests a strong down-regulation for this genotype. The evaluated DEGs corresponded to different functional categories such as antioxidant metabolism (e.g PvPer22), transcription factors (e.g *PvWRKY51*) and cell signalling (e.g *PvRPK-LRR*) which confirmed the MapMan and AgriGO results.

## 5 Conclusion

In this study, we conducted a comprehensive analysis of the common bean nodule transcriptome under drought stress using Illumina sequencing technology. To the best of our knowledge, this work represents the first systematic investigation of common bean nodule gene expression during biological nitrogen fixation under adverse conditions. Our comparative expression analysis, employing three statistical algorithms (CuffDiff, DESeq1, and edgeR), in conjunction with AgriGO and MapMan analysis, revealed a notable down-regulation of numerous metabolic pathways in common bean nodules in response to drought stress. These affected pathways encompassed antioxidant metabolism and genes related to protein kinases. Intriguingly, some gene categories, including heat shock proteins (HSPs) and specific transcription factors (TFs), were up-regulated by drought stress. While a substantial number of TFs were down-regulated, our study identified three classes of TFs (MYB, WRKY, and AP2/ERF) that were up-regulated and are well-known for their roles in plant responses to various stresses. Their involvement in nodules’ responses to drought remains a subject of ongoing research. A detailed characterization of these TFs could be a promising starting point for biotechnological strategies aimed at enhancing drought tolerance in common beans and other leguminous species. Our findings suggest that, under drought stress, common bean nodules activate genes related to abiotic stress, such as HSPs and LEA protein genes, as well as specific some transcription factors. However, the majority of DEGs are repressed, indicating a down-regulation of various metabolic processes at the transcriptional level, including antioxidant activity, the transcriptional regulation of downstream genes, and events mediated by hormones. Our results shed light on the transcriptional changes occurring in common bean nodules under drought stress. This information may serve as a foundation for the development of new strategies to enhance drought tolerance in common bean when grown under biological nitrogen fixation reliance.

## Data availability statement

The datasets presented in this study can be found in online repositories. The names of the repository/repositories and accession number(s) can be found in the article/**Supplementary Material**.

## Author contributions

Hd: Writing – review & editing, Data curation, Investigation, Methodology, Software, Writing – original draft. VC: Investigation, Methodology, Writing – review & editing. DP: Investigation, Methodology, Writing – review & editing. RP: Data curation, Writing – review & editing. CM: Writing – review & editing. JS: Writing – review & editing, Conceptualization, Funding acquisition, Project administration, Supervision.

## Supplementary material

Supplementary Figure 1 RNA-Seq experiment workflow and bioinformatics pipeline. Common bean plants submitted to drought-stress were collected **①** and root-nodules’ total RNA samples from well-watered (WW) and drought-stressed (DS) plants were used send to RNA-Seq profiling **②**. After Illumina HiSeq 2000 RNA-sequencing, the bioinformatics pipeline consisted in filter the low-quality reads (Q<20) followed by trimming using the FASTX-Toolkit **③**. The high-quality reads (Q≥20) were then mapped against the *P. vulgaris* L. reference genome using the Bowtie and TopHat2 software **④**. A table of normalized of read counts was obtained using HT-Seq-count software **⑤**, and the differentially expressed genes (DEGs) were tested using three independent algorithms: DESeq1, edgeR and CuffDiff **⑥**. Only those genes concomitantly detected in the three algorithms were considered differentially expressed. In these analyses, a list of 1451 differentially expressed genes (DEGs) was obtained from Negro Argel and BAT 477 drought-stressed nodules (output data) and used for functional annotation and further downstream analyses (Gene Ontology, MapMan etc).

Supplementary Figure 2 Negro Argel and BAT 477 common bean genotypes submitted to four days after drought stress drought stress.

Supplementary Figure 3 Identification of differentially expressed genes (DEGs) using three independent statistical algorithms. Venn diagram showing the number of DEGs concomitantly identified by CuffDiff, DESeq1 and edgeR algorithms (bold values) for BAT 477 and N. Argel genotypes. A pairwise comparison for DEGs shared between two algorithms as well as the number of DEGs specifically identified by each algorithm can be visualized. Additionally, the total number of DEGs by algorithm is indicated in parentheses.

Supplementary Figure 4 Common recruitment of drought-regulated genes in nodules of the two common bean genotypes. BAT 477 and Negro Argel shared genes from the top10 highest and lowest differentially expressed genes (DEGs) in nodules submitted to drought stress are shown.

Supplementary Figure 5 Functional annotation of differentially expressed up-regulated genes (up-regulated DEGs) in BAT 477 drought-stressed nodules. Gene ontology (GO) categorization was performed after SEA analysis using **AgriGO** Software v2.0 and the results are summarized for in the Biological Process GO category.

Supplementary Figure 6 Functional annotation of differentially expressed down-regulated genes (up-regulated DEGs) in BAT 477 drought-stressed nodules. Gene ontology (GO) categorization was performed after SEA analysis using **AgriGO** Software v2.0 and the results are summarized for in the Biological Process GO category.

Supplementary Figure 7 Functional annotation of differentially expressed up-regulated genes (up-regulated DEGs) in BAT 477 drought-stressed nodules. Gene ontology (GO) categorization was performed after SEA analysis using **AgriGO** Software v2.0 and the results are summarized for Biological Process GO category.

Supplementary Figure 8 Functional annotation of differentially expressed up-regulated genes (up-regulated DEGs) in BAT 477 drought-stressed nodules. Gene ontology (GO) categorization was performed after SEA analysis using **AgriGO** Software v2.0 and the results are summarized for Molecular Function GO category.

Supplementary Figure 9 Functional annotation of differentially expressed down-regulated genes (down-regulated DEGs) in BAT 477 (a) and Negro Argel (b) drought-stressed nodules. Gene ontology (GO) categorization was performed after SEA analysis using **AgriGO** Software v2.0 and the results are summarized for Molecular Function GO category.

Supplementary Figure 10 BAT 477 nodules regulation overview under drought stress.

Supplementary Figure 11 N. Argel nodules regulation overview under drought stress.

Supplementary Figure 12 Evaluation of expression stability candidate reference genes in common bean nodules. Three common bean candidate reference genes (*PvIDE*, *PvEf1-Alpha* and *PvAct*) were evaluated by two different algorithms (geNorm and NormFinder softwares) and used for qPCR data normalization.

Supplementary Table 1 Description of candidate reference genes and their primer sequences. Prior to DEGs validation experiments, the expression stability of three candidate reference genes was evaluated using geNorm and NormFinder algorithms in common bean (P. vulgaris L.) nodules submitted to drought stress.
