# Supplementary material for: Unraveling the drought-responsive transcriptomes in nodules of two common bean genotypes during biological nitrogen fixation
Source: Front Plant Sci. 2024 Jan 26;15:1345379. doi: 10.3389/fpls.2024.1345379 (PMC10853390; doi:10.3389/fpls.2024.1345379)
Supplement: Supplementary Figure 1 — RNA-Seq experiment workflow and bioinformatics pipeline. Common bean plants submitted to drought-stress were collected ① and root-nodules’ total RNA samples from well-watered (WW) and drought-stressed (DS) plants were used send to RNA-Seq profiling ②. After Illumina HiSeq 2000 RNA-sequencing, the bioinformatics pipeline consisted in filter the low-quality reads (Q<20) followed by trimming using the FASTX-Toolkit ③. The high-quality reads (Q≥20) were then mapped against the P. vulgaris L. reference genome using the Bowtie and TopHat2 software ④. A table of normalized of read counts was obtained using HT-Seq-count software ⑤, and the differentially expressed genes (DEGs) were tested using three independent algorithms: DESeq1, edgeR and CuffDiff ⑥. Only those genes concomitantly detected in the three algorithms were considered differentially expressed. In these analyses, a list of 1451 differentially expressed genes (DEGs) was obtained from Negro Argel and BAT 477 drought-stressed nodules (output data) and used for functional annotation and further downstream analyses (Gene Ontology, MapMan etc). [file DataSheet_1.doc]

***Supplementary Material***

**1 Supplementary Figure**


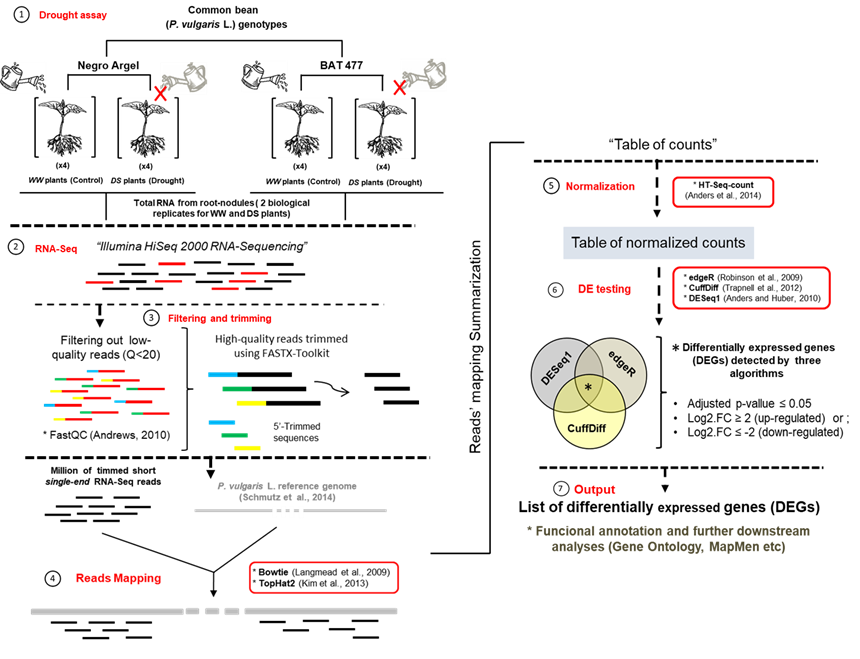


**Supplementary Figure** **S1.** RNA-Seq experiment workflow and bioinformatics pipeline. Common bean plants submitted to drought-stress were collected **①** and root-nodules’ total RNA samples from well-watered (WW) and drought-stressed (DS) plants were used send to RNA-Seq profiling **②**. After Illumina HiSeq 2000 RNA-sequencing, the bioinformatics pipeline consisted in filter the low-quality reads (Q<20) followed by trimming using the FASTX-Toolkit **③**. The high-quality reads (Q≥20) were then mapped against the *P. vulgaris* L. reference genome using the Bowtie and TopHat2 software **④**. A table of normalized of read counts was obtained using HT-Seq-count software **⑤**, and the differentially expressed genes (DEGs) were tested using three independent algorithms: DESeq1, edgeR and CuffDiff **⑥**. Only those genes concomitantly detected in the three algorithms were considered differentially expressed. In these analyses, a list of 1451 differentially expressed genes (DEGs) was obtained from Negro Argel and BAT 477 drought-stressed nodules (output data) and used for functional annotation and further downstream analyses (Gene Ontology, MapMan etc).


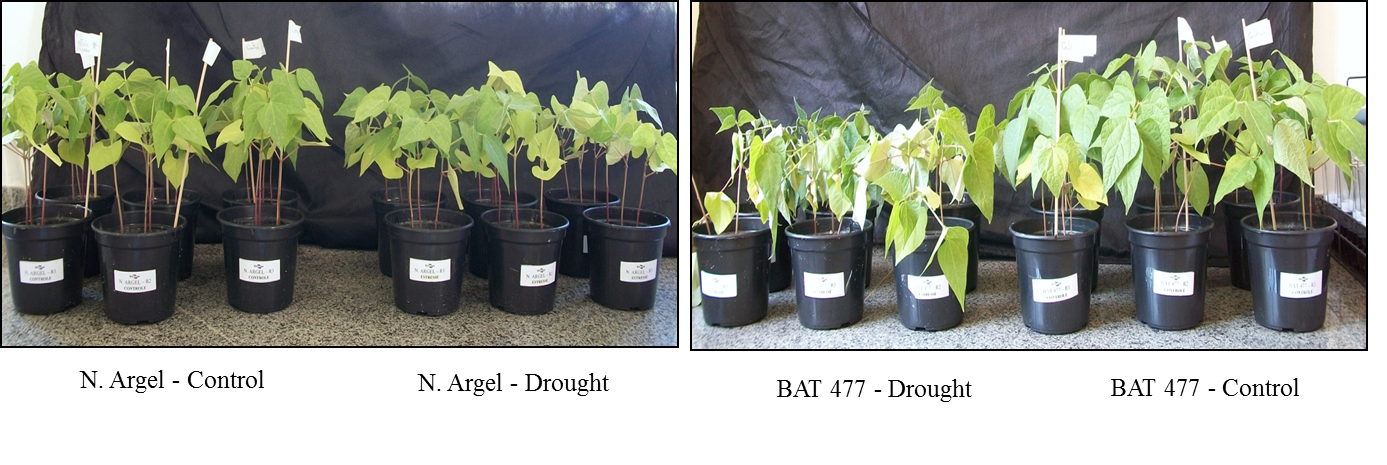


**Supplementary Figure S2.** Negro Argel and BAT 477 common bean genotypes submitted to four days after drought stress drought stress.

**
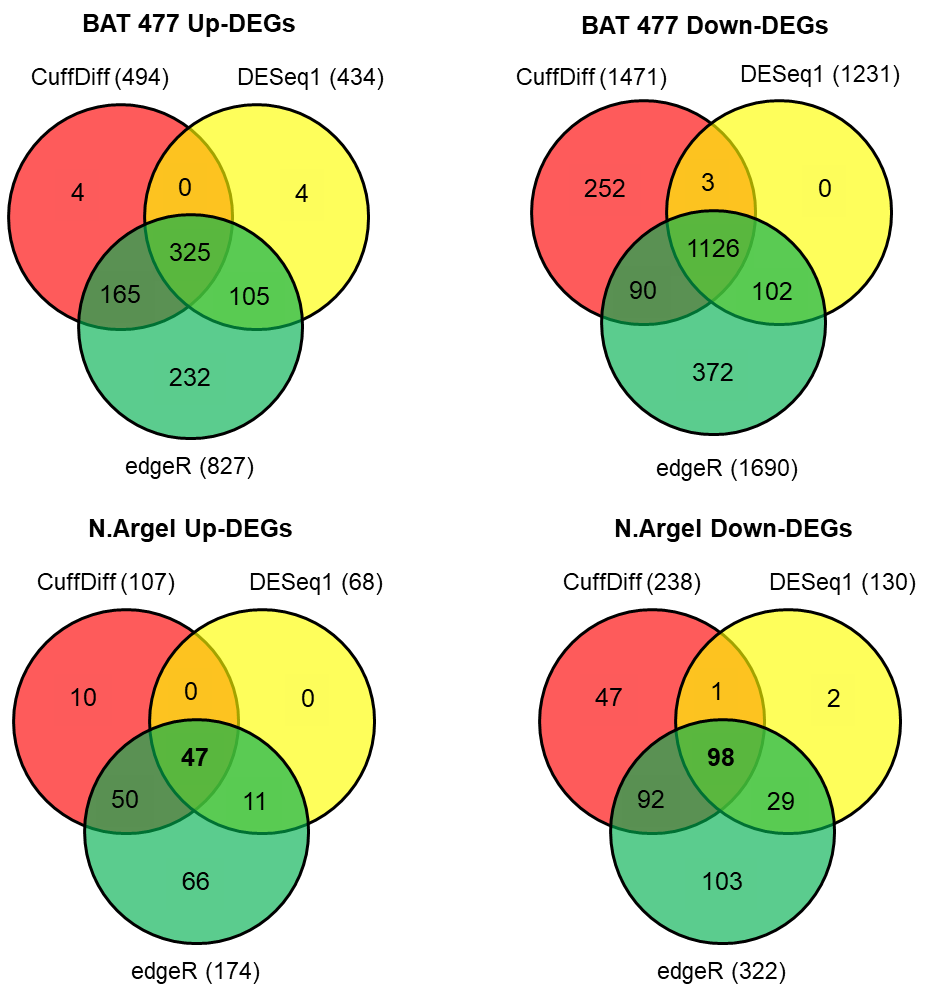
**

**Supplementary Figure S3.** Identification of differentially expressed genes (DEGs) using three independent statistical algorithms. Venn diagram showing the number of DEGs concomitantly identified by CuffDiff, DESeq1 and edgeR algorithms (bold values) for BAT 477 and N. Argel genotypes. A pairwise comparison for DEGs shared between two algorithms as well as the number of DEGs specifically identified by each algorithm can be visualized. Additionally, the total number of DEGs by algorithm is indicated in parentheses.


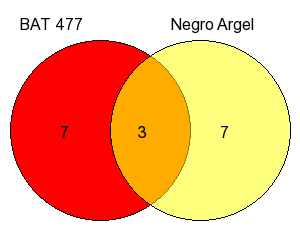

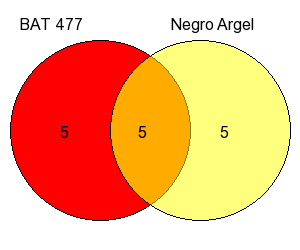


Up-DEGs

Down-DEGs

| **ID *P. vulgaris*** | **ID *M. truncatula*** | | **Description** | **E-value** | **BAT477-Log2FC** | | **N.Argel-Log2FC** |
| --- | --- | --- | --- | --- | --- | --- | --- |
| **Common Up-DEGs** | | | | | | | |
| **Phvul.008G186200.v1.0** | | Medtr5g067740.1 | hypothetical protein | 2E-57 | 8.3 | 5.95621 | |
| **Phvul.010G155300.v1.0** | | Medtr4g010320.1 | hsp20/alpha crystallin family protein | E-121 | 7.9 | 5.83528 | |
| **Phvul.009G131000.v1.0** | | Medtr3g088675.1 | chalcone and stilbene synthase family protein | 0.0 | 7.9 | 4.66107 | |
| **Phvul.001G223700.v1.0** | | Medtr7g111850.1 | galactinol synthase | 0.0 | 7.8 | 4.9781 | |
| **Phvul.001G142000.v1.0** | | Medtr7g093170.1 | seed maturation protein | 7E-53 | 7.3 | 4.23859 | |
| **Common Down-DEGs** | | | | | | | |
| **Phvul.001G128500.v1.0** | | Medtr4g076570.1 | glycoside hydrolase family 17 protein | 3E-67 | -9.5 | -6.1 | |
| **Phvul.006G075700.v1.0** | | Medtr3g467600.1 | peroxidase family protein | 0.0 | -8.3 | -3.5 | |
| **Phvul.010G122000.v1.0** | | Medtr7g117495.2 | RNA-binding (RRM/RBD/RNP motif) family protein | 3E-70 | -7.6 | -3.6 | |

**Supplementary Figure S4. Common recruitment of drought-regulated genes in nodules of the two common bean genotypes.** BAT 477 and Negro Argel shared genes from the top10 highest and lowest differentially expressed genes (DEGs) in nodules submitted to drought stress are shown.


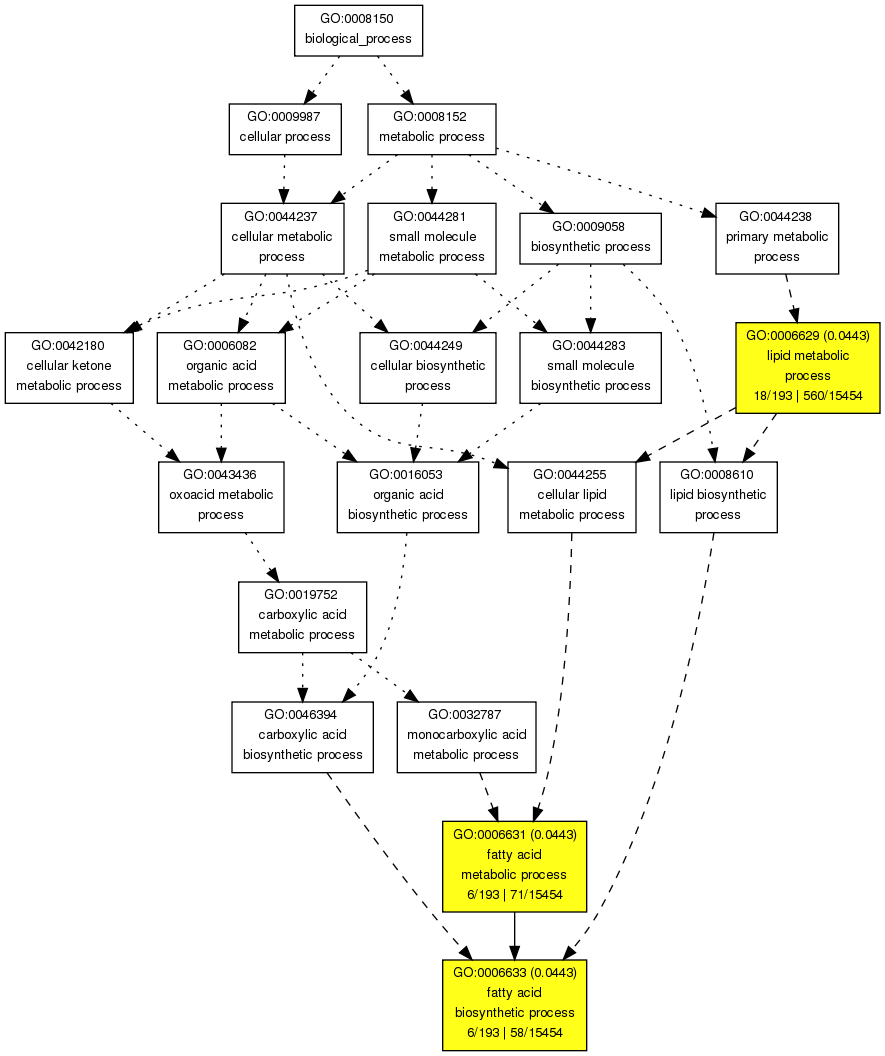


**Supplementary Figure S5.** Functional annotation of differentially expressed up-regulated genes (up-regulated DEGs) in BAT 477 drought-stressed nodules. Gene ontology (GO) categorization was performed after SEA analysis using **AgriGO** Software v2.0 and the results are summarized for in the Biological Process GO category.


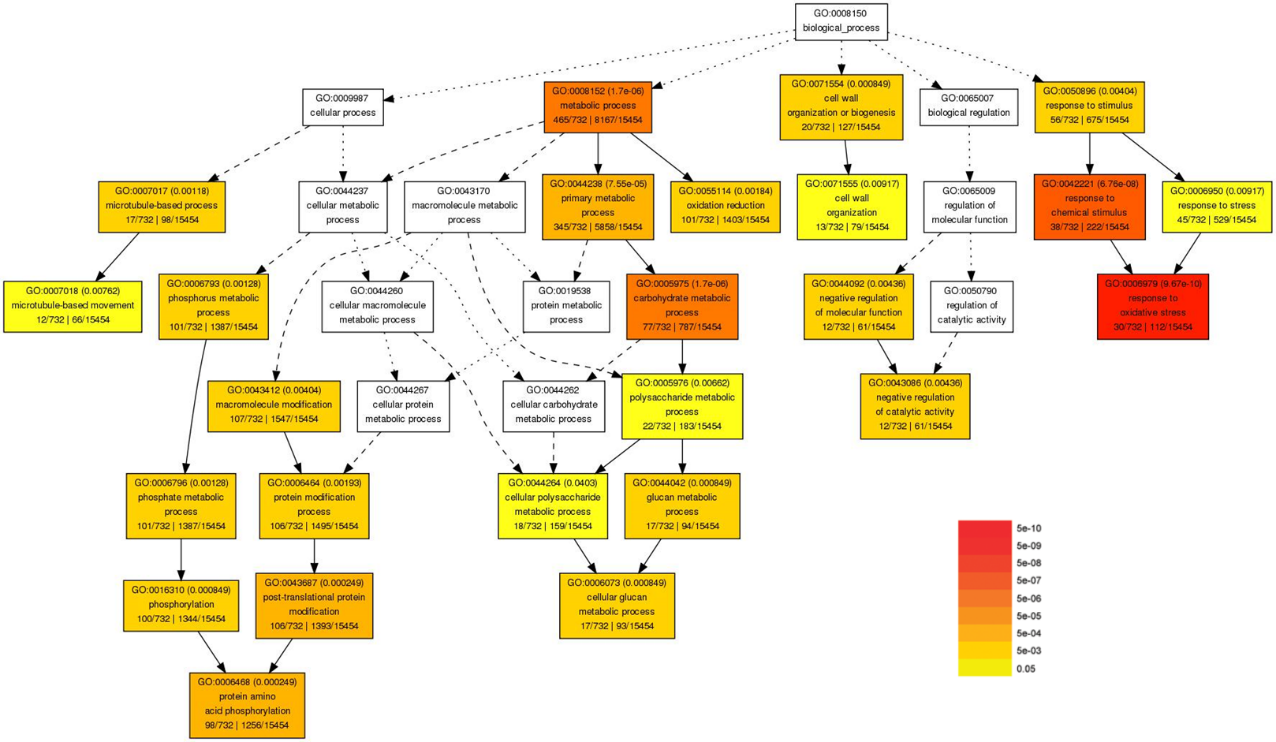


**Supplementary Figure S6.** Functional annotation of differentially expressed down-regulated genes (up-regulated DEGs) in BAT 477 drought-stressed nodules. Gene ontology (GO) categorization was performed after SEA analysis using **AgriGO** Software v2.0 and the results are summarized for in the Biological Process GO category.


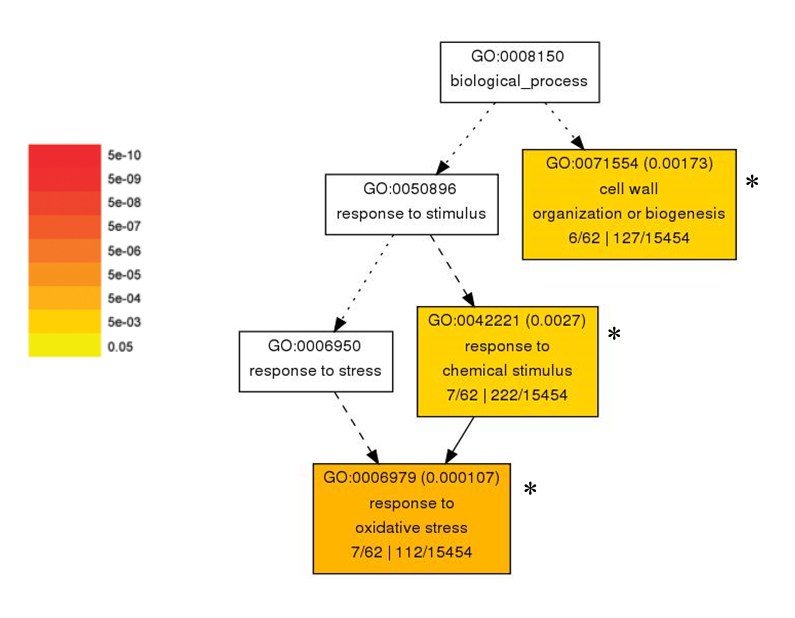


**Supplementary Figure S7.** Functional annotation of differentially expressed up-regulated genes (up-regulated DEGs) in BAT 477 drought-stressed nodules. Gene ontology (GO) categorization was performed after SEA analysis using **AgriGO** Software v2.0 and the results are summarized for Biological Process GO category.

**
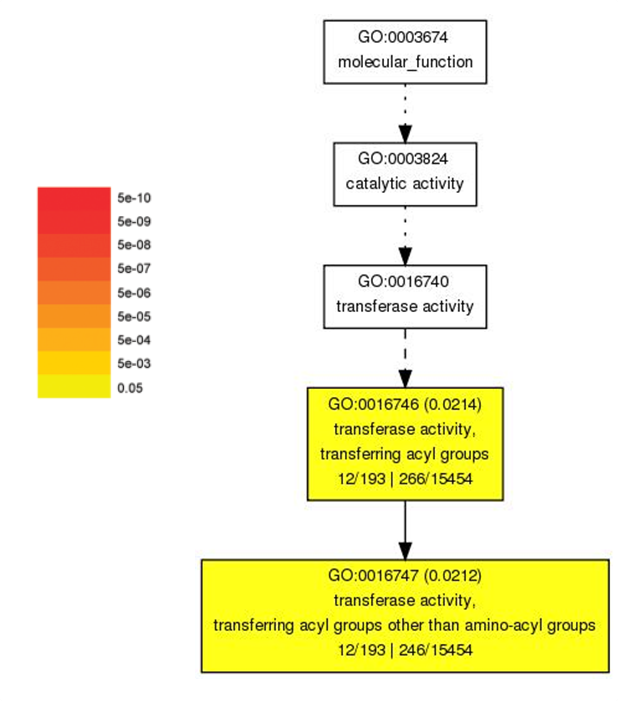
**

**Supplementary Figure S8.** Functional annotation of differentially expressed up-regulated genes (up-regulated DEGs) in BAT 477 drought-stressed nodules. Gene ontology (GO) categorization was performed after SEA analysis using **AgriGO** Software v2.0 and the results are summarized for Molecular Function GO category.

**Supplementary Figure S9.** Functional annotation of differentially expressed down-regulated genes (down-regulated DEGs) in BAT 477 (a) and Negro Argel (b) drought-stressed nodules. Gene ontology (GO) categorization was performed after SEA analysis using **AgriGO** Software v2.0 and the results are summarized for Molecular Function GO category.


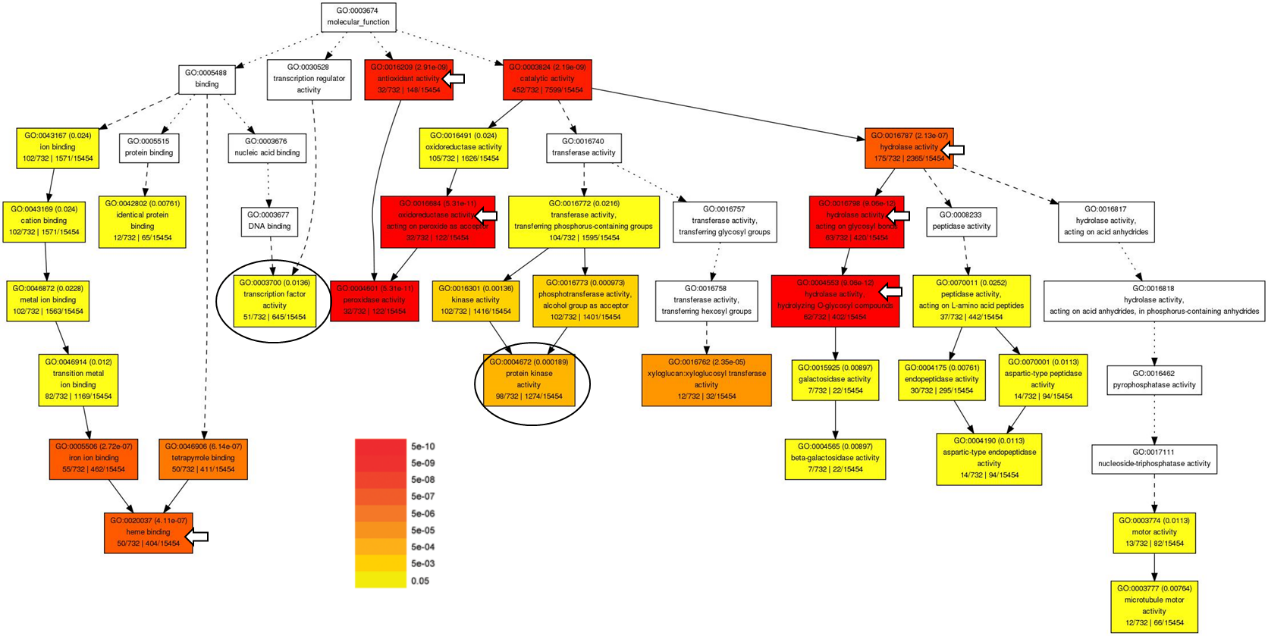

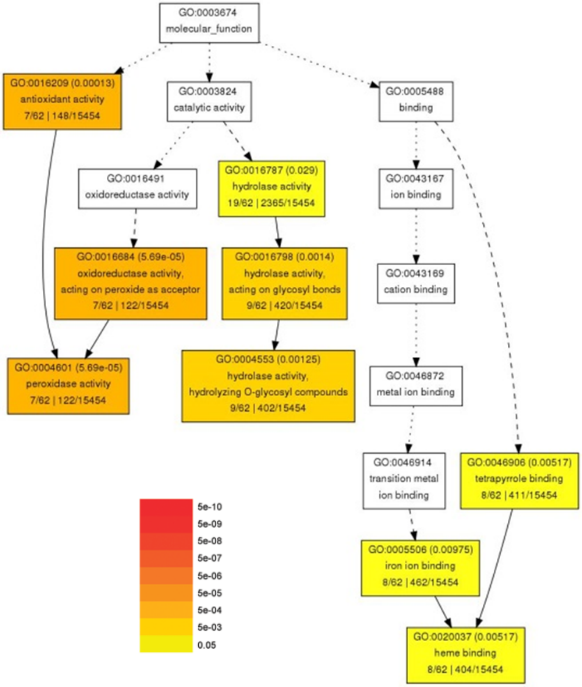

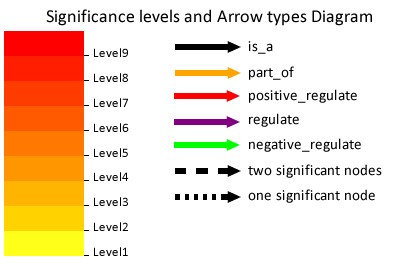


a

b


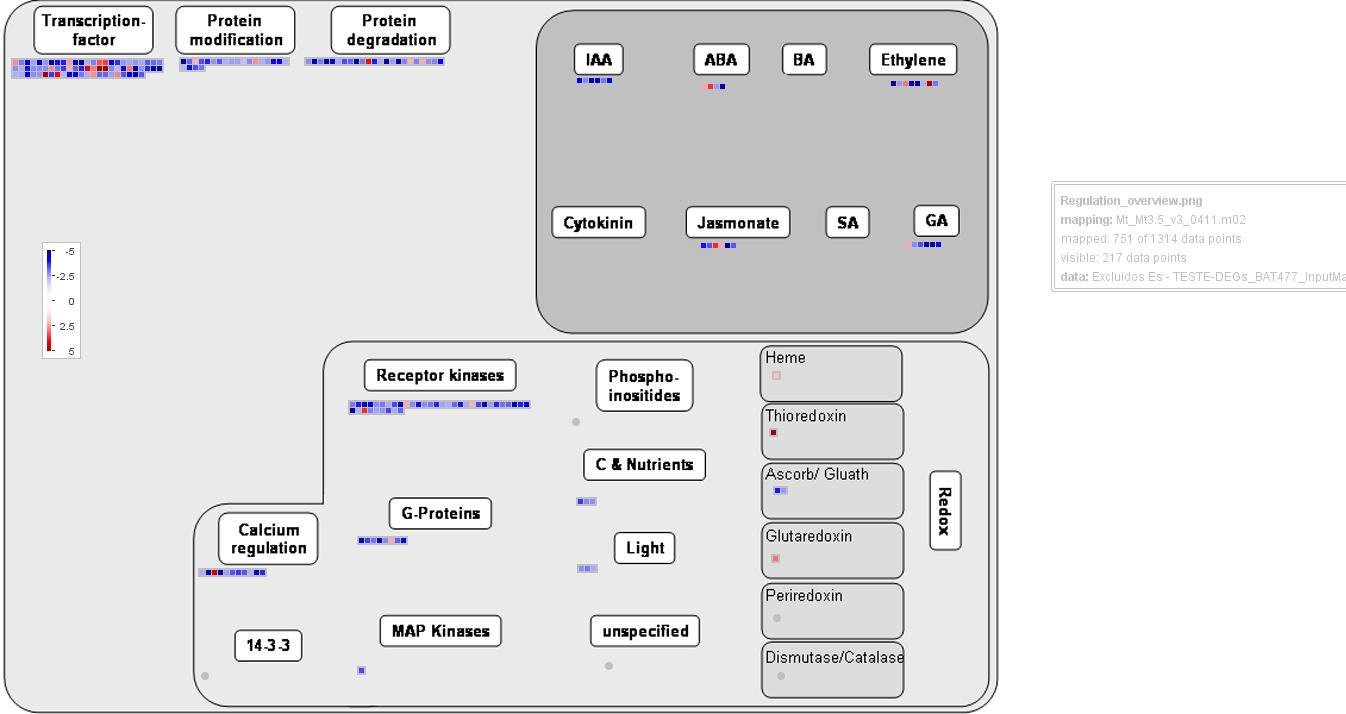


**Supplementary Figure S10. BAT 477 nodules regulation overview under drought stress.**


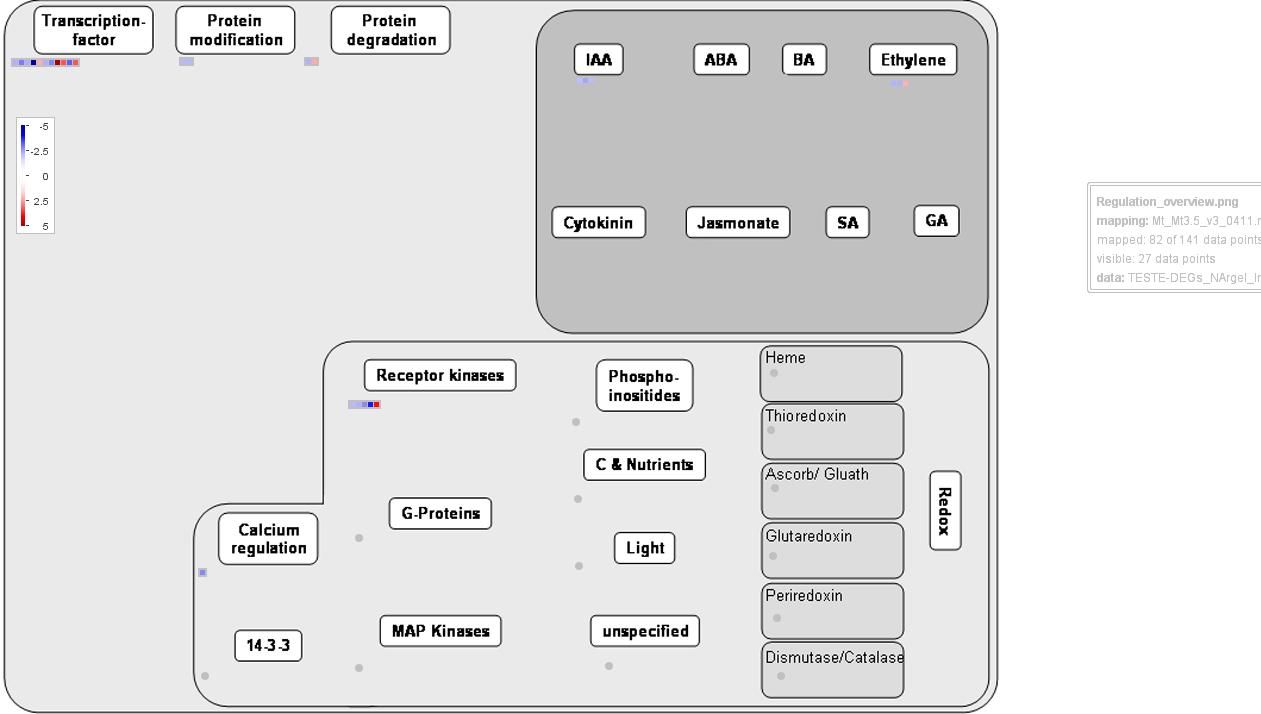


**Supplementary Figure S11** **N. Argel nodules regulation overview under drought stress.**

A) geNorm

| 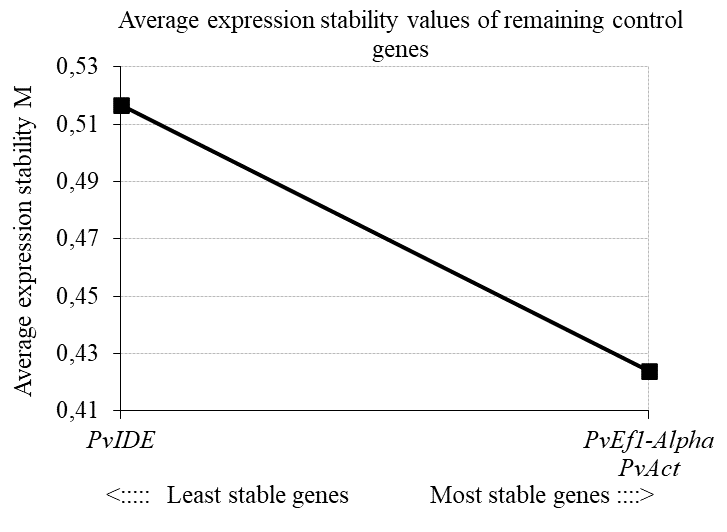  B) NormFinder |
| --- |
| 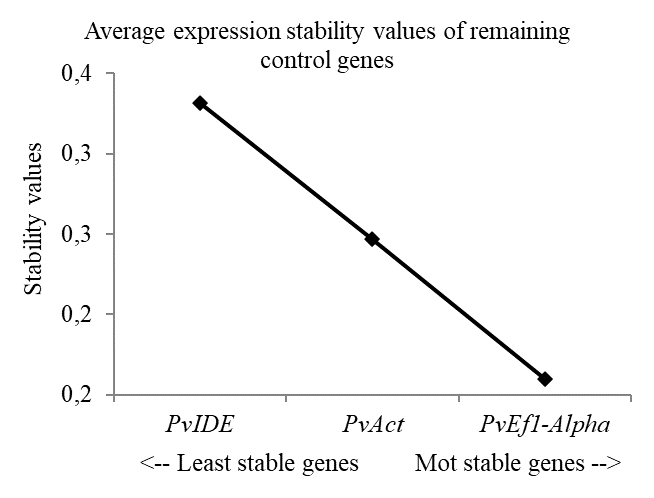 |

**Supplementary Figure S12. Evaluation of expression stability candidate reference genes in common bean nodules.** Three common bean candidate reference genes (*PvIDE*, *PvEf1-Alpha* and *PvAct*) were evaluated by two different algorithms (geNorm and NormFinder softwares) and used for qPCR data normalization.
