# Supplementary material for: Unraveling the drought-responsive transcriptomes in nodules of two common bean genotypes during biological nitrogen fixation
Source: Front Plant Sci. 2024 Jan 26;15:1345379. doi: 10.3389/fpls.2024.1345379 (PMC10853390; doi:10.3389/fpls.2024.1345379)
Supplement: Supplementary Table 1 — Description of candidate reference genes and their primer sequences. Prior to DEGs validation experiments, the expression stability of three candidate reference genes was evaluated using geNorm and NormFinder algorithms in common bean (P. vulgaris L.) nodules submitted to drought stress. [file Table_1.doc]

**Supplemental Table S1** **Description of candidate reference genes and their primer sequences.** Prior to DEGs validation experiments, the expression stability of three candidate reference genes was evaluated using geNorm and NormFinder algorithms in common bean (P. vulgaris L.) nodules submitted to drought stress.

| **Gene Name** | ***GeneBank*** | | | **Function** | **Primers’ sequences (5’ 🡪 3’)** | | **Eff. ± SD** | |  |
| --- | --- | --- | --- | --- | --- | --- | --- | --- | --- |
| Candidate Reference Genes | | | | | | | | |  |
| *Actin*  (*PvAct*) | | *PvAct 2*Parcial CDS (GenBank: [EU581898.1](http://www.ncbi.nlm.nih.gov/nuccore/EU581898.1)) | A major component of the plant cell cytoskeleton | | | AGCTCAGCCGTTGAGAAGAG  ATGGATGGCTGGAACAGAAC | | 1.85±0.002 | |
| Elongation Factor 1-Alfa (*PvEF1*-*Alfa*) | | *PvEF1-Alfa* Parcial CDS (GenBank: [EF660340.1](http://www.ncbi.nlm.nih.gov/nuccore/EF660340.1)) | Protein that associates with ribosomes during the elongation phase of protein synthesis | | | AGGCTGATTGTGCTGTCCTT  ACACCAAGGGTGAAAGCAAG | | 1.88±0.003 | |
| (*PvIDE*) | | *PvIDE* (GenBank:[FE702602.1](http://www.ncbi.nlm.nih.gov/nucest/FE702602.1)) | Insulin degrading enzyme | | | GCAACCAACCTTTCATCAGC  AGAAATGCCTCAACCCTTTG | | 1.86±0.004 | |
